# Synaptotagmin-1 enables frequency coding by suppressing asynchronous release in a temperature dependent manner

**DOI:** 10.1038/s41598-019-47487-9

**Published:** 2019-08-05

**Authors:** Vincent Huson, Maaike A. van Boven, Alexia Stuefer, Matthijs Verhage, L. Niels Cornelisse

**Affiliations:** 1 Department of Functional Genomics, Clinical Genetics, Center for Neurogenomics and Cognitive Research, Amsterdam University Medical Center- Location VUmc, Amsterdam, The Netherlands; 2 Department of Functional Genomics, Center for Neurogenomics and Cognitive Research, VU University Amsterdam, Amsterdam, The Netherlands

**Keywords:** Molecular neuroscience, Synaptic vesicle exocytosis

## Abstract

To support frequency-coded information transfer, mammalian synapses tightly synchronize neurotransmitter release to action potentials (APs). However, release desynchronizes during AP trains, especially at room temperature. Here we show that suppression of asynchronous release by Synaptotagmin-1 (Syt1), but not release triggering, is highly temperature sensitive, and enhances synchronous release during high-frequency stimulation. In Syt1-deficient synapses, asynchronous release increased with temperature, opposite to wildtype synapses. Mutations in Syt1 C2B-domain polybasic stretch (Syt1 K326Q,K327Q,K331Q) did not affect synchronization during sustained activity, while the previously observed reduced synchronous response to a single AP was confirmed. However, an inflexible linker between the C2-domains (Syt1 9Pro) reduced suppression, without affecting synchronous release upon a single AP. Syt1 9Pro expressing synapses showed impaired synchronization during AP trains, which was rescued by buffering global Ca^2+^ to prevent asynchronous release. Hence, frequency coding relies on Syt1’s temperature sensitive suppression of asynchronous release, an aspect distinct from its known vesicle recruitment and triggering functions.

## Introduction

The frequency of action potential (AP) firing is one of the important characteristics of spike trains by which information is encoded in the brain^1–4^. In the hippocampus, firing rate has been found to encode receptive fields for spatial information^5^, time^6,7^, and items^8^, and is generally important for episodic memory^9^. To support rate coding for a wide range of frequencies, most central synapses maintain fast synaptic transmission during sustained activity^2^, with basal firing rates exceeding 100 Hz^10^, and even higher burst firing rates^11^.

Whereas the mechanisms involved in the fast synaptic response to a single AP stimulation are well characterized^12^, it is still unclear how the additional demands for synaptic transmission during sustained activity are met. Previous studies show that synchronous release in response to a single AP is hardly affected by changes in temperature between room- and body-temperature^13–16^. However, during sustained activity at high frequencies, synaptic vesicle release becomes highly asynchronous when the temperature is lowered^14^. This suggests a temperature sensitive mechanism that synchronizes release during high frequency stimulation, distinct from release triggering itself. Propositions for this include changes in postsynaptic current^15^, decreased^14,17^ or increased^18,19^ release probability, and increased synchronization of the Ca^2+^ signal with the AP through faster currents^13,15,20^ or enhanced clearance^14,21^. This latter possibility is in line with previous findings that buffering global Ca^2+^ inside the nerve terminal, and the resulting suppression of asynchronous release, promotes synchronous release. The fact that synchronous release is promoted at the expense of asynchronous release, suggests competition between the two^22^. However, whether any of the proposed mechanisms are involved in synchronisation of release during sustained high-frequency activity remains unclear.

One factor that is still poorly understood is the role of the fast Ca^2+^-sensor Synaptotagmin-1 (Syt1). Syt1 has been shown to be essential for triggering synchronous vesicle release^23,24^, and is also involved in vesicle recruitment and docking^25–28^, as well as regulating vesicle endocytosis after vesicle release^29–33^. However, beyond promoting synchronous release and recruiting vesicles, it is well established that, at rest, Syt1, or its functionally-related paralog Syt2, exerts an inhibitory or ‘clamping’ effect on spontaneous release^34–36^ and suppresses the asynchronous release component that follows the synchronous spike^37–39^. These two phenomena have been related to inhibition of a second Ca^2+^-sensor by Syt1, representing a common mechanism for both^39,40^. Although little is currently known about Syt1’s release inhibition during high-frequency activity, given the competition between synchronous and asynchronous release, directed suppression of asynchronous release by Syt1’s inhibitory function might play an important role in maintaining synchronous release during sustained high-frequency stimulation.

In this study, we investigate the mechanisms underlying temperature-dependent synchronization of neurotransmission during high-frequency stimulation in hippocampal autaptic excitatory neurons. Our results suggest that increased synchronization of presynaptic Ca^2+^ cannot explain increased synchronous release at the end of high-frequency train stimulation. Instead, we characterize a temperature sensitive, inhibitory function of Syt1 acting in maintaining synchronous release during high-frequency activity. We conclude that besides the previously reported triggering and vesicle recruitment functions of Syt1, suppression of asynchronous release is an additional mechanism through which Syt1 synchronizes release. This function appears essential for sustaining frequency coded signalling, thus allowing neurons to transmit information over a broad frequency bandwidth.

## Results

### Frequency-coding is highly temperature sensitive

**Figure 1:**
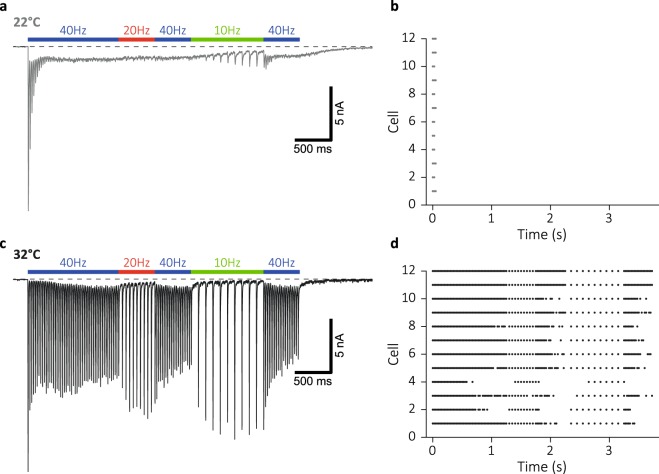
Raising temperature enables frequency-coded signalling during sustained transmission. (**a**) Typical EPSCs evoked by a 150-AP train with variable frequencies of 40, 20 and 10 Hz in autaptic hippocampal neurons (wild type) at room temperature (22 ± 1 °C) and (**c**) near-physiological temperature (32 ± 1 °C). (**b**) Raster plot of cells firing above 30% amplitude of first evoked response at 22 °C and (**d**) 32 °C.

At rest or during low-frequencies, synaptic responses are synchronized to APs and relatively insensitive to temperature changes, whereas they rapidly desynchronize during high-frequency stimulation at room temperature (RT). To assess the effect of temperature on frequency-coded information transfer, we compared synaptic responses to a 150-AP train with variable frequencies (40, 20 and 10 Hz) at 22.0 ± 1 °C and 32.5 ± 1 °C (hereafter 22 °C and 32 °C, respectively) in single hippocampal neurons cultured on glia micro-islands (autapses)^41^. With a threshold for post-synaptic spike detection at 30% amplitude of the first evoked response, neurons at 22 °C failed to transmit after the first three pulses. The remaining neurotransmission was dominated by asynchronous release, and homogeneous at different frequencies, constituting a loss of information transfer (Fig. 1a,b). However, when measured at 32 °C, synchronous responses persisted (Fig. 1c), clearly marking the different stimulation frequencies (Fig. 1d). A similar result was reached with a lower threshold at 10%, or a higher threshold at 50% of the first evoked response (Supplementary Fig. S1). Thus, a 10 °C change in temperature critically changes the efficacy of frequency-coding in hippocampal synapses.

### Synchronous responses to single action potentials are not temperature sensitive

**Figure 2:**
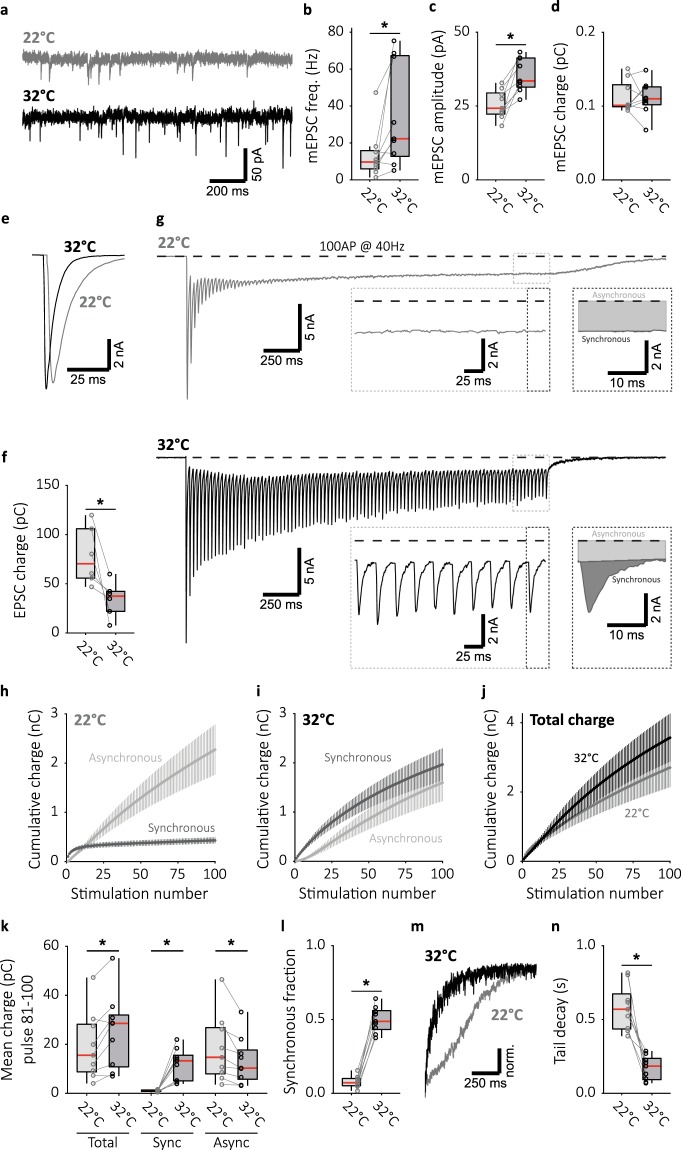
Raising temperature synchronizes neurotransmission during sustained stimulation and suppresses asynchronous release. (**a**) Representative traces of mEPSC recordings at 22 °C (top) and 32 °C (bottom). Boxplots showing (**b**) mEPSC frequency (22 °C: 9.655 ± 5.34 Hz, n = 9; 32 °C: 22.27 ± 14.1 Hz, n = 9), (**c**) mEPSC amplitude (22 °C: 24.25 ± 3.20 pA, n = 9; 32 °C: 33.50 ± 5.05 pA, n = 9), and (**d**) mEPSC charge at 22 °C and 32 °C (22 °C: 0.1010 ± 0.00363 pC, n = 9; 32 °C: 0.1099 ± 0.0139 pC, n = 9). (**e**) Single EPSC representative traces and (**f**) boxplot of charge transferred per EPSC at 22 °C and 32 °C (22 °C: 70.34 ± 19.0 pC, n = 6; 32 °C: 78.15 ± 12.0 pC, n = 6). (**g**) Representative traces of 40 Hz train stimulation (100 pulses, 2.5 s) at 22 °C and 32 °C. The expanded traces illustrate steady state release dynamics, and single pulse zooms display division synchronous and asynchronous release. (**h**) Cumulative plots (mean ± S.E.M.) of charge transferred synchronously and asynchronously at 22 °C and (**i**) 32 °C. (**j**) Cumulative total charge (mean ± S.E.M.) at 22 °C and 32 °C. (**k**) Boxplots with late-train charge averaged over the final 20 pulses of the 40 Hz train, for paired 22 °C and 32 °C recordings. Displayed for total charge (22 °C: 15.53 ± 8.21 pC, n = 9; 32 °C: 28.54 ± 6.83 pC, n = 9), and synchronous (22 °C: 1.135 ± 0.293 pC, n = 9; 32 °C: 13.27 ± 5.134 Hz, n = 9) and asynchronous charge (22 °C: 14.69 ± 8.50 Hz, n = 9; 32 °C: 10.29 ± 5.92 Hz, n = 9) separately. (**l**) Boxplot with fraction of late-train charge transfer during the final 20 pulses released synchronously (22 °C: 0.07162 ± 0.0239, n = 9; 32 °C: 0.4872 ± 0.0658, n = 9). (**m**) Typical traces of asynchronous tail release, normalized to peak release. (**n**) Boxplot with decay tau from single exponential fits of asynchronous tail release at 22 °C and 32 °C (22 °C: τ = 0.5694 ± 0.129 s, n = 9; 32 °C: τ = 0.1843 ± 0.0854 s, n = 9). (*p < 0.05, Wilcoxon signed-rank test).

We investigated which aspects of synaptic transmission were temperature sensitive. We measured the effect of temperature on spontaneous synaptic release and observed a more than 2-fold higher rate of miniature excitatory post-synaptic currents (mEPSCs) at 32 °C (Fig. 2a,b). Furthermore, mEPSC amplitudes were increased at 32 °C (Fig. 2c), while mEPSC charge remained unchanged (Fig. 2d). Excitatory post-synaptic currents (EPSCs) after a single AP remained synchronous, both at 22 °C and 32 °C (Fig. 2e). In fact, more charge was transferred at 22 °C (Fig. 2f), in line with previous observations^14^, although the amplitude remained unchanged (data not shown). We conclude that synchronous responses evoked by a single AP are temperature insensitive, while spontaneous release is inhibited at 22 °C.

### Raising temperature increases late-train synchronous release at the cost of asynchronous release

Synchronization of synaptic responses is most likely due to synchronization of the presynaptic vesicle release process. One possible cause for the temperature dependent loss of synchronous responses during high frequency stimulation is increased competition from asynchronous release for the releasable pool of vesicles^22^. To investigate this, we sought an objective way of estimating synchronous and asynchronous release during high-frequency stimulation, under the assumption that most asynchronous release is blocked by buffering global Ca^2+^ ^22,42,43^. We compared synaptic responses, scaled to their peak amplitude, before (naive) and after application of the slow Ca^2+^ buffer EGTA-AM, and noticed that asynchronous release occurred during the decay phase of the EPSC (green area, Supplementary Fig. S2a). Contribution of NMDA currents to this component were considered negligible due to high (4 mM) extracellular Mg^2+^. We determined for each cell the “synchronous time window”, defined as the time interval for which the asynchronous release within the interval (green shaded area, Supplementary Fig. S2a) balances the synchronous release outside the interval (red shaded area, Supplementary Fig. 2a), and found a median value of 22.65 ± 5.65 ms (Supplementary Fig. S2b, n = 18). To prevent underestimation of the synchronous component and overestimation of the asynchronous component, we set the synchronous time window for our analysis to 25 ms. The standing current due to asynchronous release during high-frequency stimulation, which persists beyond the length of an individual pulse, was subtracted at the start of each pulse before calculating the synchronous component (Supplementary Fig. S2c). While we noticed that in the presence of EGTA-AM the EPSC width did not change considerably throughout the train, it is possible that the asynchronous contribution within the synchronous window is higher at the end of the train. This would slightly overestimate synchronous release but prevents erroneous conclusions about release desynchronization.

Quantifying synchronous release during 100AP 40 Hz train stimulation at 22 °C we observed that synchronous release rapidly decayed, leaving asynchronous as the dominant form of release (Fig. 2g,h). Synchronous release at the end of the train stimulation (late-train synchronous release), quantified by averaging the charge of the last 20 responses in the train, was negligible (Fig. 2k). In contrast, at 32 °C synchronous release persisted until the end of the train (Fig. 2g,i,k). This more than 10-fold increase was specific for synchronous release as total release only doubled and asynchronous release decreased (Fig. 2h–k). Expressed as a fraction of total release, the synchronous component (late-train synchronous fraction) was increased almost 7 fold (Fig. 2l). In addition to decreased asynchronous release during late-train stimulation, asynchronous release after the stimulation train (tail release), decayed much faster at 32 °C, as determined by a mono-exponential fit (Fig. 2m,n). All temperature effects were reversible (Supplementary Fig. S3). Taken together these results indicate an inverse relationship between synchronous and asynchronous release, where an increase in synchronous release at 32 °C seemingly comes at the cost of asynchronous release. This is in line with the previous conclusion that synchronous and asynchronous release compete for the same vesicle pool^22^. As such the loss of late-train synchronous release at 22 °C can be explained by a shift towards asynchronous release.

### Buffering global Ca^2+^ synchronizes late-train release but reduces total release

Next, we set out to investigate the mechanism by which competition between synchronous and asynchronous release during train stimulation shifts to synchronous release at 32 °C. Previous studies suggested that faster intracellular calcium dynamics play a role, by increasing Ca^2+^ clearance, by reducing Ca^2+^ influx, or by both^13,14^. To investigate this, we examined release at RT during high-frequency stimulation before and after bath application of EGTA-AM. This slow calcium buffer is known to prevent build-up of global intracellular Ca^2+^ ^44–46^ and synchronize release at RT^22^. Release triggered by a single AP was reduced in EGTA-AM (Supplementary Fig. S4a,b). However, during 40 Hz stimulation a strong synchronous component remained in contrast to untreated cells (Supplementary Fig. S4c–e,g). Meanwhile, late-train asynchronous release decreased (Supplementary Fig. S4g), leading to an almost 10-fold increase in the synchronous fraction of late-train release (Supplementary Fig. S4h), similar to our observations at 32 °C (Fig. 2l). We conclude that buffering of global Ca^2+^, suppresses asynchronous release and synchronizes late-train release, in line with previous studies^22,38^.

Besides promoting asynchronous release, global intracellular Ca^2+^ also triggers other processes in the presynapse, such as accelerated refilling of the readily releasable vesicle pool (RRP)^47–50^. After application of EGTA-AM, total release was decreased by more than 50% (Supplementary Fig. S4f,g), in contrast to the 100% increase at 32 °C (Fig. 2k). Therefore, either global intracellular Ca^2+^ is indeed reduced but the loss of Ca^2+^-dependent RRP refilling is outweighed by faster Ca^2+^-independent RRP refilling at 32 °C ^14^, or a process other than increased Ca^2+^ clearance synchronizes release at 32 °C.

### Raising temperature does not suppress asynchronous release in Syt1 deficient synapses

**Figure 3:**
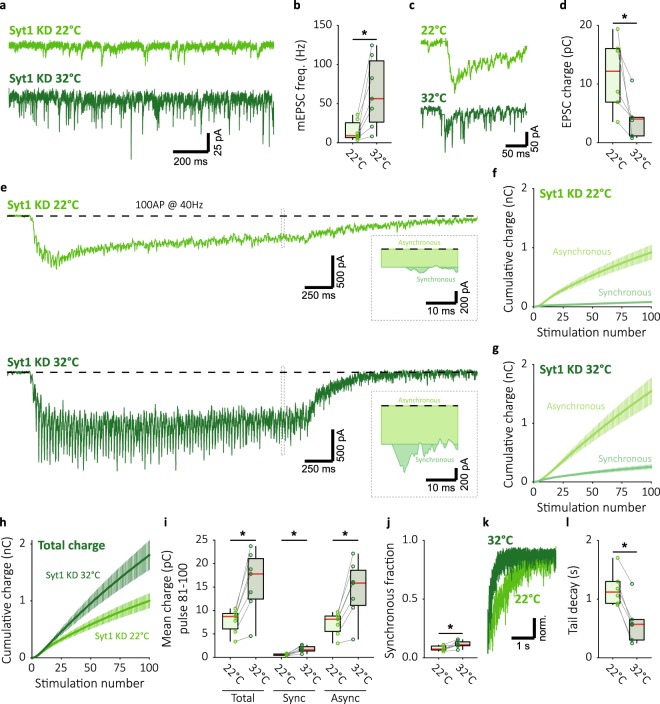
Raising temperature in Synaptotagmin-1 deficient synapses enhances asynchronous release instead of suppressing it. (**a**) Representative traces of mEPSC recordings at 22 °C (top) and 32 °C (bottom), and (**b**) boxplot showing mEPSC frequency (22 °C: 9.002 ± 3.09 Hz, n = 7; 32 °C: 56.38 ± 35.7 Hz, n = 7). (**c**) Single EPSC representative traces and (**d**) boxplot of charge transferred per EPSC at 22 °C and 32 °C (22 °C: 12.19 ± 4.57 pC, n = 6; 32 °C: 4.037 ± 1.59 pC, n = 6). (**e**) Representative traces of 40 Hz train stimulation (100 pulses, 2.5 s) at 22 °C and 32 °C; single pulse zooms display division synchronous and asynchronous release. (**f**) Cumulative plots (mean ± S.E.M.) of charge transferred synchronously and asynchronously at 22 °C and (**g**) 32 °C. (**h**) Cumulative total charge (mean ± S.E.M.) at 22 °C and 32 °C. (**i**) Boxplots with late-train charge averaged over the final 20 pulses of the 40 Hz train, for paired 22 °C and 32 °C recordings. Displayed for total charge (22 °C: 8.711 ± 0.905 pC, n = 7; 32 °C: 17.79 ±  ± 4.03 pC, n = 7), and synchronous (22 °C: 0.5791 ± 0.157 pC, n = 7; 32 °C: 1.594 ± 0.353 pC, n = 7) and asynchronous charge (22 °C: 8.132 ± 1.17 pC, n = 7; 32 °C: 15.84 ± 3.57 pC, n = 7) separately. (**j**) Boxplot with fraction of late-train charge transfer during the final 20 pulses released synchronously (22 °C: 0.07414 ± 0.00782, n = 7; 32 °C: 0.1104 ± 0.00939, n = 7). (**k**) Typical traces of asynchronous tail release, normalized to peak release. (**l**) Boxplot with decay tau from single exponential fits of asynchronous tail release at 22 °C and 32 °C (22 °C: τ = 1.120 ± 0.186 s, n = 6; 32 °C: τ = 0.5694 ± 0.174 s, n = 6). (*p < 0.05, Wilcoxon signed-rank test).

In order to establish if at 32 °C low levels of global intracellular Ca^2+^ cause suppression of asynchronous release, we investigated the effect of temperature in Syt1 knock-down (KD) synapses. Inhibition of Syt1 expression eliminates synchronous release^23,24^, and previous work has shown that EGTA-AM abolishes the remaining asynchronous release^38^. Therefore, this component is also expected to be reduced at 32 °C in these synapses, if increasing temperature lowers levels of global intracellular Ca^2+^. In Syt1 KD synapses, the effect of temperature on spontaneous release and single EPSCs was comparable to that observed in wild-type synapses (Fig. 3a–d and Fig. 2a-f). Median mEPSC frequency was decreased at 22 °C (Fig. 3a,b), while EPSC charge was increased (Fig. 3c,d). In accordance with previous reports^23,24^, Syt1 KD synapses lacked synchronous release during 40 Hz stimulation (Fig. 3e,f,i). At 32 °C, a small but significant increase in synchronous release was observed, although asynchronous release remained dominant (Fig. 3e,g,i). As a consequence, the late-train synchronous fraction of release was low (Fig. 3j). Total release at 32 °C was double that at 22 °C (Fig. 3h,i), almost all of which was due to an increase in the asynchronous component. In contrast, after stimulation, asynchronous tail release decayed faster to baseline at 32 °C (Fig. 3k,l). This faster decay at 32 °C in Syt1 KD synapses suggests that Ca^2+^ clearance is faster at 32 °C^14,21^. Nevertheless, the increase in asynchronous release suggests that during high frequency stimulation at 32 °C Ca^2+^ influx outweighs increased Ca^2+^ clearance, and a considerable global intracellular Ca^2+^ component remains. Therefore, we conclude that mechanisms other than global intracellular Ca^2+^ regulation synchronize release during high frequency stimulation at 32 °C in WT synapses. While likely some endogenous Syt1 remains in Syt1 KD synapses, the reversal in temperature dependence of asynchronous release compared with WT synapses (Fig. 2), indicates a clear role for Syt1 in this process.

### Syt1 D363N inhibits and delays the onset of asynchronous release

**Figure 4:**
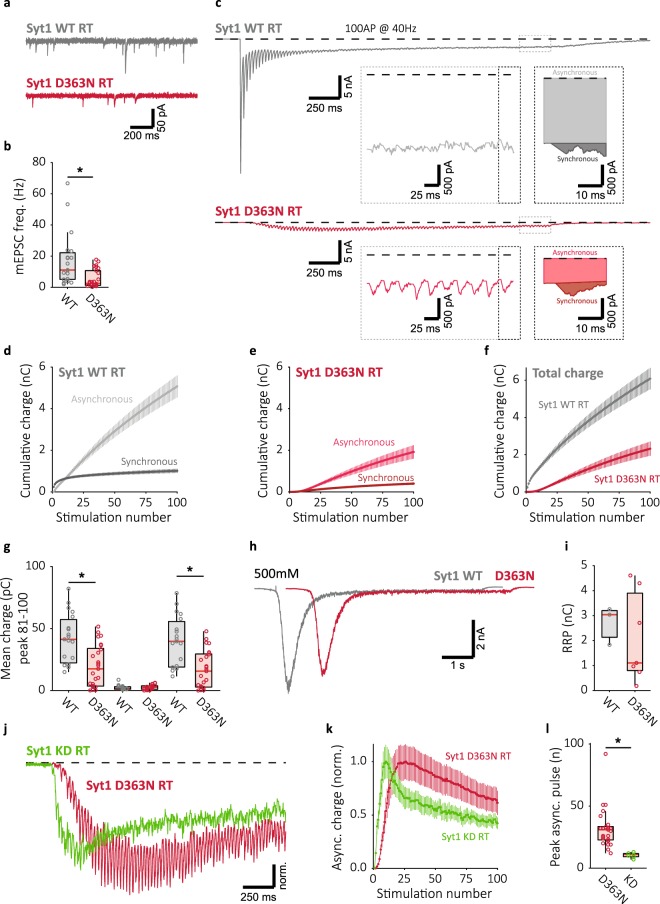
Deficient Ca^2+^ binding in Syt1 D363N mutant suppresses all forms of release and delays the onset of the asynchronous component. (**a**) Representative traces of mEPSC recordings from Syt1 KO neurons rescued with WT (top) or D363N mutant (bottom) constructs, and (**b**) boxplot showing mEPSC frequency (Syt1 WT: 11.11 ± 7.85 Hz, n = 19; Syt1 D363N: 2.585 ± 1.82 Hz, n = 26). (**c**) Representative traces of 40 Hz train stimulation (100 pulses, 2.5 s) in Syt1 WT or D363N expressing synapses. The expanded traces illustrate steady state release dynamics, and single pulse zooms display division synchronous and asynchronous release. (**d**) Cumulative plots (mean ± S.E.M.) of charge transferred synchronously and asynchronously in Syt1 WT, or (E) Syt1 D363N expressing synapses. (**f**) Cumulative total charge (mean ± S.E.M.) in Syt1 WT and D363N expressing synapses. (**g**) Boxplots with late-train charge averaged over the final 20 pulses of the 40 Hz train, for Syt1 WT and D363N expressing synapses. Displayed for total charge (Syt1 WT: 41.30 ± 18.3 pC, n = 19; Syt1 D363N: 17.51 ± 14.5 pC, n = 25), and synchronous (Syt1 WT: 1.703 ± 0.676 pC, n = 19; Syt1 D363N: 2.651 ± 1.404 pC, n = 25) and asynchronous charge (Syt1 WT: 39.64 ± 19.9 pC, n = 19; Syt1 D363N: 15.61 ± 13.0 pC, n = 25) separately. (**h**) Representative traces of 500 mM hypertonic sucrose responses, and (**i**) boxplot of RRP charge estimates for Syt1 WT and D363N expressing synapses (Syt1 WT: 3.039 ± 0.219 nC, n = 3; Syt1 D363N: 1.105 ± 0.923 nC, n = 7). (**j**) Representative traces of 40 Hz train stimulation in Syt1 D363N rescue and Syt1 KD synapses, scaled to the peak asynchronous current. (**k**) Asynchronous charge plot (mean ± S.E.M.), normalized to peak mean charge for Syt1 D363N rescue and Syt1 KD synapses. (**l**) Boxplot of pulse number containing the peak asynchronous charge for Syt1 D363N rescue and Syt1 KD synapses (Syt1 D363N: 31 ± 7, n = 25; Syt1 KD: 11 ± 1, n = 7). All recordings at room temperature (RT), ~22 °C unmonitored. (*p < 0.05, Wilcoxon rank sum test).

Syt1 and Syt2 have previously been reported to suppress spontaneous^34–36^ and asynchronous release^37–39,51,52^. Given the proposed competition between synchronous and asynchronous release for synaptic vesicles^22^, this may represent a mechanism for Syt1 to establish the temperature dependent shift from asynchronous to synchronous release we observe. To investigate this possibility, we set out to test whether Syt1’s release inhibitory function could suppress asynchronous release under conditions of elevated global Ca^2+^ induced by our stimulation paradigm. Mutating a single negatively charged amino acid in the Syt1 C2B Ca^2+^-binding loop (D363N) both abolishes synchronous release and suppresses asynchronous release^37,39^. However, this result is not unambiguous as a similar mutation (D309A, D363A, D365A) enhances spontaneous release^53^. To confirm release inhibition by the Syt1 D363N mutation at rest, and examine its effect on Syt1’s inhibitory function during high-frequency stimulation, we expressed either Syt1 D363N, or Syt1 WT in hippocampal autapses. To avoid mixing of mutant Syt1 with any residual endogenous Syt1, these constructs were expressed in neurons derived from homozygous Syt1 knock out (KO) mice. Recording at RT, we found that Syt1 D363N reduced mEPSC frequency compared to Syt1 WT rescue (Fig. 4a,b), in contrast to reports with the D309A, D363A, D365A mutations^53^. Furthermore, while Syt1 WT expressing synapses showed typical evoked release, Syt1 D363N strongly reduced both synchronous, asynchronous, and total release during 40 Hz train stimulation (Fig. 4c–f). Additionally, Syt1 D363N expressing synapses showed reductions in late-train total and asynchronous release of more than 50% compared to Syt1 WT, while late-train synchronous release was negligible for both (Fig. 4g). Nevertheless, the RRP was similar between Syt1 WT and D363N expressing synapses as measured with 500 mM hypertonic sucrose (Fig. 4h,i). Finally, the remaining asynchronous component in Syt1 D363N expressing synapses was delayed in onset compared to Syt1 KD synapses (Fig. 4j,k), reaching its maximum 20 APs later (Fig. 4l). This indicates that, in addition to its lack of fusion triggering, Syt1 D363N has a net inhibitory effect on vesicle fusion, both at rest and during repetitive stimulation, highlighting a potential native function of Syt1 for suppressing non-synchronous release during high-frequency stimulation, separate from its action in synchronous release triggering.

### Normal late-train synchronous release in Syt1 3K expressing synapses

**Figure 5:**
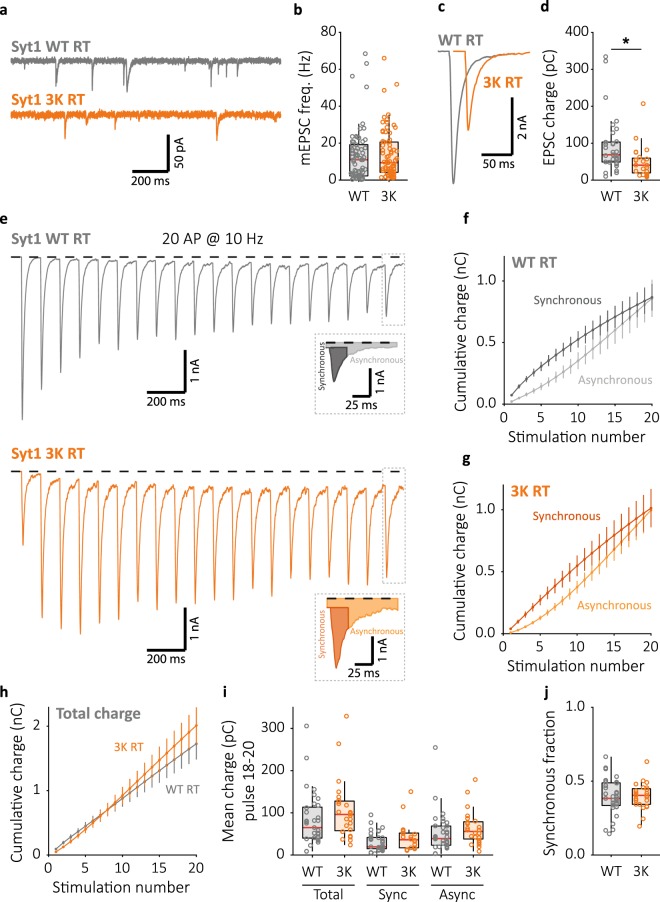
Syt1 3K expressing synapses show impaired first evoked response but have normal release inhibition and late train synchronous release. (**a**) Representative traces of mEPSC recordings from Syt1 KO neurons rescued with WT (top) or 3K mutant (bottom) constructs, and (**b**) boxplot showing mEPSC frequency (Syt1 WT: 10.97 ± 8.72 Hz, n = 70; Syt1 3 K: 9.388 ± 6.42 Hz, n = 72). (**c**) Single EPSC representative traces and (**d**) boxplot of charge transferred per EPSC in Syt1 WT and 3 K expressing synapses (Syt1 WT: 68.81 ± 28.6 pC, n = 33; Syt1 3 K: 40.53 ± 19.5 pC, n = 24). (**e**) Representative traces of 10 Hz train stimulation (20 pulses, 2 s) in Syt1 WT or 3K expressing synapses; single pulse zooms display division synchronous and asynchronous release. (**f**) Cumulative plots (mean ± S.E.M.) of charge transferred synchronously and asynchronously in Syt1 WT, or (**g**) Syt1 3K expressing synapses. (**h**) Cumulative total charge (mean ± S.E.M.) in Syt1 WT and 3K expressing synapses. (**i**) Boxplots with late-train charge averaged over the final 3 pulses of the 10 Hz train, for Syt1 WT and 3K expressing synapses. Displayed for total charge (Syt1 WT: 65.09 ± 29.1 pC, n = 33; Syt1 3K: 96.07 ± 34.9 pC, n = 24), and synchronous (Syt1 WT: 19.41 ± 36.3 pC, n = 33; Syt1 3K: 36.28 ± 17.6 pC, n = 24) and asynchronous charge (Syt1 WT: 39.07 ± 19.5 pC, n = 33; Syt1 3K: 55.80 ± 21.2 pC, n = 24) separately. (**j**) Boxplot with fraction of late-train charge transfer during the final 3 pulses released synchronously (Syt1 WT: 0.3834 ± 0.0616, n = 33; Syt1 3K: 0.4038 ± 0.0522, n = 24). All recordings at room temperature (RT), ~22 °C unmonitored. (*p < 0.05, Wilcoxon rank sum test).

Given the ability of Syt1 to suppress release during high frequency stimulation, we hypothesised that this could contribute to synchronization of late-train release by influencing the competition between asynchronous and synchronous release^22^ in favour of the latter. This may constitute a mechanism for temperature dependent synchronization. To investigate this, we examined the Syt1 3K mutant, where three key amino acids of the polybasic region in the C2B domain were neutralised (K326Q, K327Q, K331Q). This is analogous to previously reported mutations in Syt2 that impair its ability to inhibit spontaneous release, leading to the suggestion that these residues are critical to Syt2’s ability to inhibit a second Ca^2+^ sensor, and implicating a possible role in supressing asynchronous release^39^. In contrast to findings in Syt2^39^, we found that at RT the mEPSC frequency was normal in hippocampal Syt1 KO autapses expressing Syt1 3K (Fig. 5a,b). However, in line with previous reports, the first evoked response was reduced (Fig. 5c,d)^39^.

Next, we assessed the competition between synchronous and asynchronous release during 10 Hz stimulation at RT in Syt1 WT and 3 K expressing synapses. At this frequency, release remained synchronous in Syt1 WT expressing synapses (Fig. 5e–f), thus allowing effects of the 3K mutations on synchronization to be observable. Despite the reduction in the first evoked response, Syt1 3K expressing synapses displayed similar synchronous release during train stimulation as Syt1 WT due to strong facilitation (Fig. 5e–g,i), and released the same or more in total (Fig. 5h,i). Asynchronous release was not different between Syt1 WT and 3K expressing synapses (Fig. 5i), and no significant difference was observed in the synchronous fraction of late-train release at 10 Hz (Fig. 5j). The results remained similar when increasing the stimulation frequency to 20 Hz, although here Syt1 3K autapses showed a slight increase in the synchronous fraction (Supplementary Fig. S5f). Application of EGTA-AM prevented facilitation of the EPSC during train stimulation in Syt1 3K expressing synapses (Supplementary Fig. S5i), with a strong trend towards lower total release compared to Syt1 WT with EGTA-AM (Supplementary Fig. S5l,m). Taken together, these findings indicate that the 3K mutations produce an overall reduction in Syt1 triggered release, that can be compensated by global intracellular Ca^2+^, rather than impaired inhibition of spontaneous and asynchronous release as shown for Syt2^39^. As such, we conclude that the Syt1 3K mutations are not suitable for testing for a possible role of Syt1’s release inhibition in temperature dependent synchronization. Instead, the observed characteristics of Syt1 3 K mimic the phenotype of a Syt1 mutant with similar mutations in the C2B polybasic region (K325A, K327A) that have previously been shown to regulate vesicle tethering to the plasma membrane in preparation for synchronous release^28^.

### Impaired release inhibition in Syt1 9Pro neurons desynchronizes late-train release

**Figure 6:**
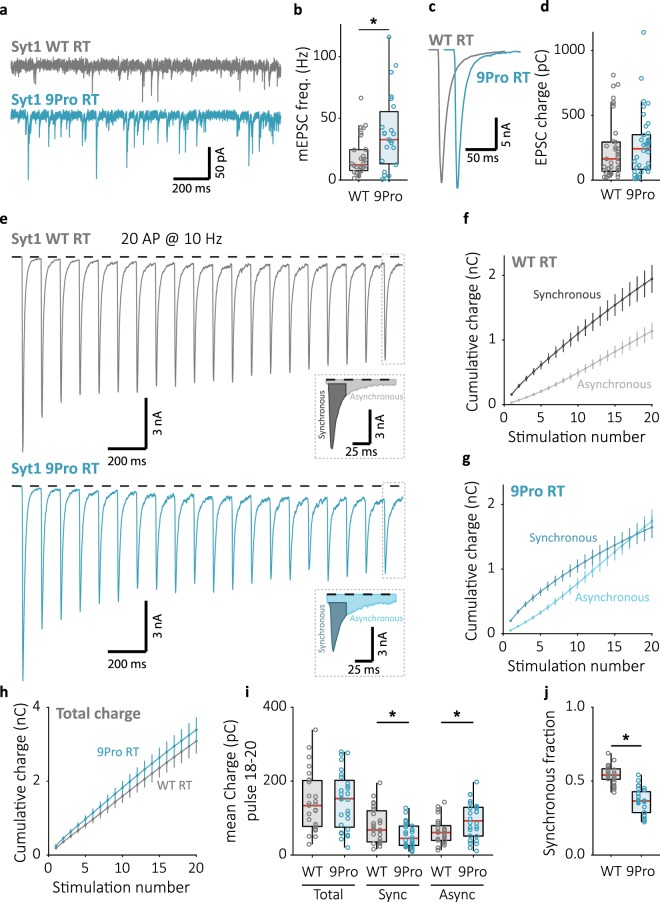
Impaired release inhibition in Syt1 9Pro expressing synapses decreases late-train synchronous release and increases asynchronous release. (**a**) Representative traces of mEPSC recordings from Syt1 KO neurons rescued with WT (top) or 9Pro mutant (bottom) constructs, and (**b**) boxplot showing mEPSC frequency (Syt1 WT: 12.33 ± 6.33 Hz, n = 28; Syt1 9Pro: 32.76 ± 20.4 Hz, n = 23). (**c**) Single EPSC representative traces and (**d**) boxplot of charge transferred per EPSC in Syt1 WT and 9Pro expressing synapses (Syt1 WT: 163.5 ± 112 pC, n = 37; Syt1 9Pro: 241.5 ± 152 pC, n = 37). (**e**) Representative traces of 10 Hz train stimulation (20 pulses, 2 s) in Syt1 WT or 9Pro expressing synapses; single pulse zooms display division synchronous and asynchronous release. (**f**) Cumulative plots (mean ± S.E.M.) of charge transferred synchronously and asynchronously in Syt1 WT, or (**G**) Syt1 9Pro expressing synapses. (**h**) Cumulative total charge (mean ± S.E.M.) in Syt1 WT and 9Pro expressing synapses. (**i**) Boxplots with late-train charge averaged over the final 3 pulses of the 10 Hz train, for Syt1 WT and 9Pro expressing synapses. Displayed for total charge (Syt1 WT: 133.8 ± 63.8 pC, n = 27; Syt1 9Pro: 152.8 ± 61.6 pC, n = 28), and synchronous (Syt1 WT: 67.66 ± 32.9 pC, n = 27; Syt1 9Pro: 45.16 ± 22.4 pC, n = 28) and asynchronous charge (Syt1 WT: 60.76 ± 21.0 pC, n = 27; Syt1 9Pro: 92.49 ± 39.10 pC, n = 28) separately. (**j**) Boxplot with fraction of late-train charge transfer during the final 3 pulses released synchronously (Syt1 WT: 0.5409 ± 0.0360, n = 27; Syt1 9Pro: 0.3654 ± 0.0755, n = 28). All recordings at room temperature (RT), ~22 °C unmonitored. (*p < 0.05, Wilcoxon rank sum test).

**Figure 7:**
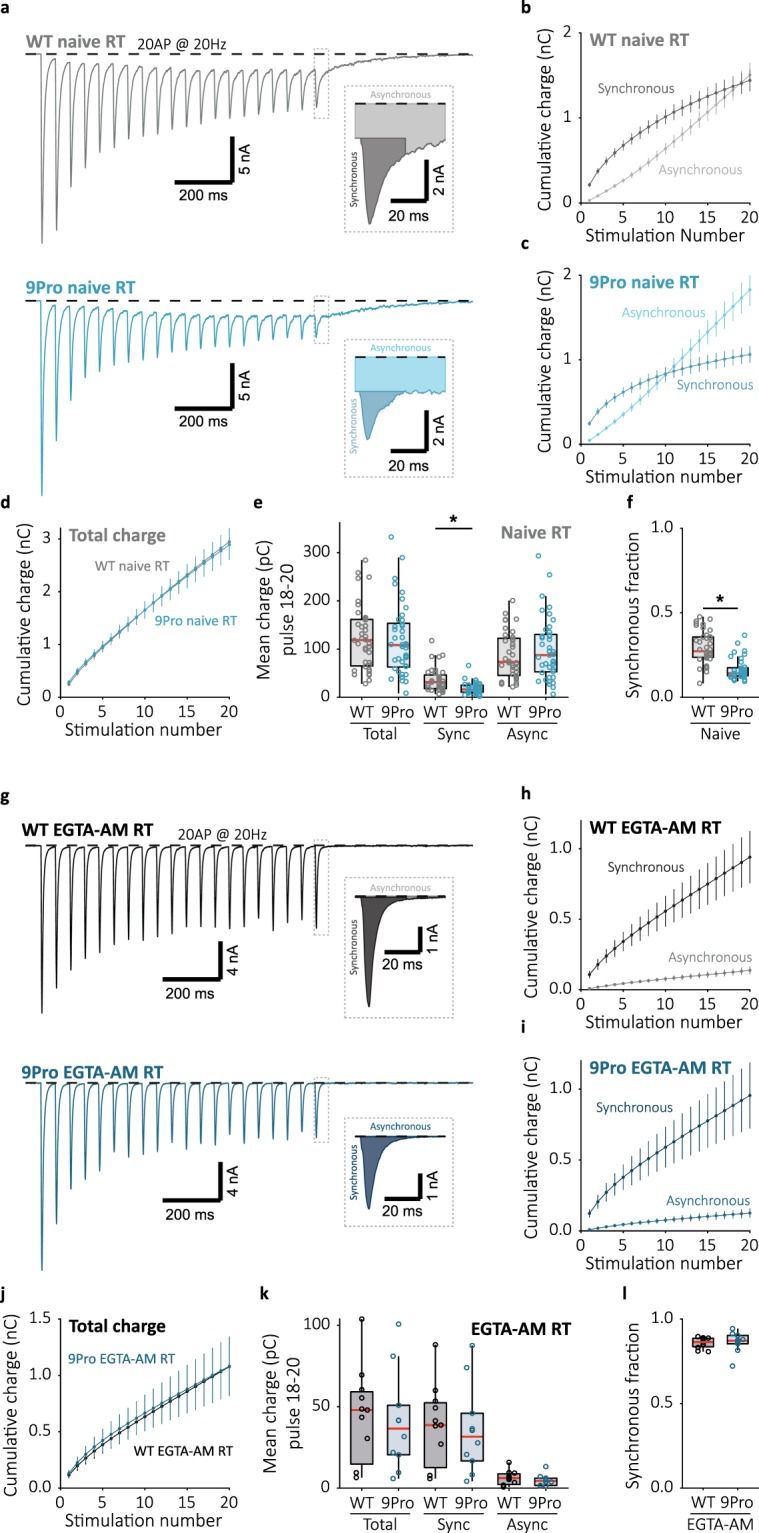
Impaired synchronization in Syt1 9Pro is rescued when global Ca^2+^ is buffered by EGTA-AM. (**a**) Representative traces of 20 Hz train stimulation (20 pulses, 1 s) in Syt1 WT or 9Pro expressing synapses in the absence (Naive) or (**g**) presence of EGTA-AM; single pulse zooms display division synchronous and asynchronous release. (**b**) Cumulative plots (mean ± S.E.M.) of charge transferred synchronously and asynchronously in naive Syt1 WT and (**h**) in the presence of EGTA-AM, or (**C**) naive Syt1 9Pro expressing synapses and (**i**) in the presence of EGTA-AM. (**d**) Cumulative total charge (mean ± S.E.M.) in naive and (**j**) EGTA-AM Syt1 WT and 9Pro conditions. (**e**) Boxplots with late-train charge averaged over the final 3 pulses of the 20 Hz train, for naive and (**k**) EGTA-AM Syt1 WT and 9Pro conditions. Displayed for total charge (WT Naive: 118.8 ± 46.3 pC, n = 40; 9Pro Naive: 108.2 ± 44.4 pC, n = 41; WT EGTA-AM: 47.93 ± 17.5 pC, n = 11; 9Pro EGTA-AM: 36.53 ± 15.39 pC, n = 10), and synchronous (WT Naive: 31.47 ± 13.5 pC, n = 40; 9Pro Naive: 16.28 ± 6.84 pC, n = 41; WT EGTA-AM: 38.70 ± 14.7 pC, n = 11; 9Pro EGTA-AM: 31.59 ± 14.6 pC, n = 10)and asynchronous charge (WT Naive: 73.23 ± 39.2 pC, n = 40; 9Pro Naive: 87.43 ± 39.2 pC, n = 41; WT EGTA-AM: 6.145 ± 3.08 pC, n = 11; 9Pro EGTA-AM: 4.383 ± 2.16 pC, n = 10) separately. (**f**,**l**) Boxplot with fraction of late-train charge transfer during the final 3 pulses released synchronously for naive and EGTA-AM conditions respectively (WT Naive: 0.2728 ± 0.0592, n = 40; 9Pro Naive: 0.1426 ± 0.0195, n = 41; WT EGTA-AM: 0.8634 ± 0.0243, n = 11; 9Pro EGTA-AM: 0.8718 ± 0.0245, n = 10). All recordings at room temperature (RT), ~22 °C unmonitored. (*p < 0.05, Wilcoxon rank sum test).

As an alternative candidate for investigating the role of inhibition by Syt1 in temperature dependent synchronization of late-train release, we selected the Syt1 9Pro mutant, in which the flexible linker between the C2 domains is replaced with a rigid nine-residue proline segment. This mutant was reported to show impaired inhibition of spontaneous release while the first evoked was unaffected^54,55^. Although effects from these mutations on asynchronous release have not yet been investigated, the specific orientation of Syt1’s C2 domains enforced by the rigid proline linker in Syt1 9Pro is believed to affect Syt1’s ability to clamp fusion in general^55^, making this a possible location for the competitive advantage of synchronous release observed at higher temperatures. In line with this, we observed significantly higher mEPSC frequencies in hippocampal Syt1 KO autapses expressing Syt1 9Pro than in Syt1 WT rescue recorded at RT (Fig. 6a,b). The first evoked response was not different (Fig. 6c,d), as shown previously^54,55^. Interestingly, during 10 Hz trains, synchronous release decreased to a greater extent in Syt1 9Pro expressing synapses, and asynchronous release increased concomitantly (Fig. 6e–g,i). As a consequence, the synchronous fraction of late-train release was significantly reduced compared to Syt1 WT (Fig. 6j), without affecting total release during 10 Hz stimulation (Fig. 6h,i). Desynchronization in Syt1 9Pro expressing synapses became even more pronounced at 20 Hz (Fig. 7a–c,e,f). Hence, although a similar number of vesicles is released during train stimulation in Syt1 WT and 9Pro expressing synapses, release in Syt1 9Pro is less synchronous and more asynchronous.

Besides increased competition from asynchronous release, poor synchronization in Syt1 9Pro expressing synapses might be due to an overall deficit in triggering of late-train synchronous release. To investigate this, we suppressed asynchronous release at RT by buffering global intracellular Ca^2+^ with EGTA-AM. After incubation with EGTA-AM, release completely synchronized, similar to Syt1 WT expressing synapses with EGTA-AM (Fig. 7g–i,k,l). Application of EGTA-AM reduced the first evoked response and the total release during the train to a similar extent for Syt1 WT and Syt1 9Pro expressing synapses (Supplementary Fig. S7; Fig. 7d,j). We conclude that late-train synchronous release in Syt1 9Pro expressing synapses is only impaired when asynchronous release is present. Likely, in Syt1 9Pro expressing synapses, decreased suppression of asynchronous release allows it to out-compete synchronous release for synaptic vesicles.

Given a role for Syt1 in suppressing asynchronous release, this would likely also affect the decay of asynchronous tail release after stimulation trains. However, asynchronous tail release after 100 pulse trains at 10 Hz recorded at RT was similar in Syt1 9Pro and Syt1 WT expressing synapses (Supplementary Fig. S6). However, in Syt1 9Pro expressing synapses the decay of tail release slowed significantly when the stimulation frequency was increased up to 40 Hz, but not in Syt1 WT (Supplementary Fig. S6b). Possibly, only when intracellular Ca^2+^ remains high for an extended period (as after 40 Hz), can an effect from Syt1’s release inhibitory function be detected in asynchronous tail release.

### Suppression of asynchronous release by Syt1 is required for temperature dependent synchronization

**Figure 8:**
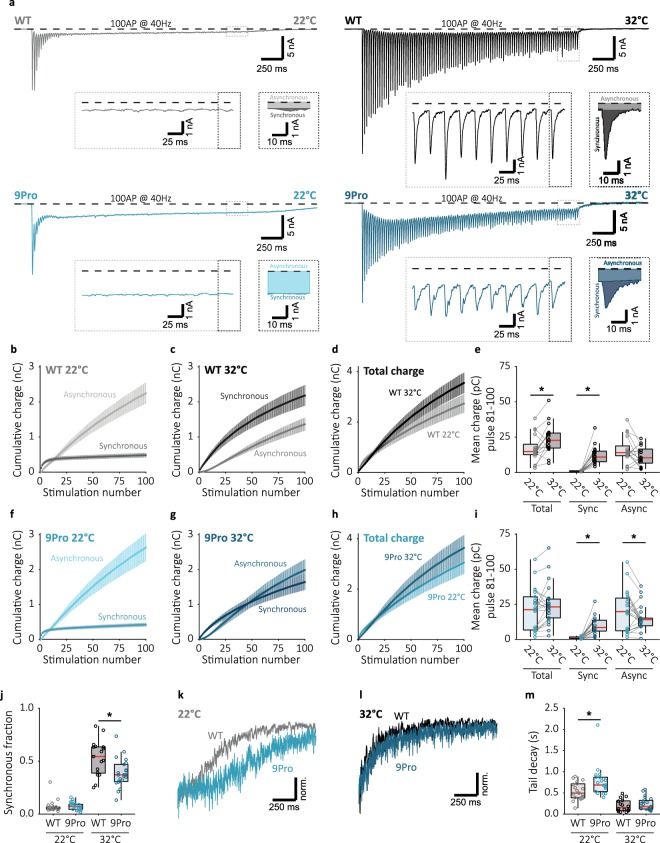
Impaired release inhibition Syt1 9Pro reduces the temperature dependent increase in synchronization. (**a**) Representative traces of 40 Hz train stimulation (100 pulses, 2.5 s) at 22 °C and 32 °C, for Syt1 WT and 9Pro expressing synapses. The expanded traces illustrate steady state release dynamics, and single pulse zooms display division synchronous and asynchronous release. (**b**,**f**) Cumulative plots (mean ± S.E.M.) of charge transferred synchronously and asynchronously in Syt1 WT and 9Pro respectively at 22 °C and (**c**,**g**) 32 °C. (**d**,**h**) Cumulative total charge (mean ± S.E.M.) at 22 °C and 32 °C in Syt1 WT and 9Pro expressing synapses respectively. (**e**,**i**) Boxplots with late-train charge averaged over the final 20 pulses of the 40 Hz train, for paired 22 °C and 32 °C recordings in Syt1 WT and 9Pro respectively. Displayed for total charge (WT 22 °C: 14.75 ± 2.79 pC; WT 32 °C: 22.73 ± 5.41 pC, n = 17; 9Pro 22 °C: 21.19 ± 13.1 pC; 9Pro 32 °C: 23.19 ± 5.90 pC, n = 22), and synchronous (WT 22 °C: 0.8044 ± 0.246 pC; WT 32 °C: 10.89 ± 3.92 pC, n = 17; 9Pro 22 °C: 0.9444 ± 0.364 pC; 9Pro 32 °C: 8.504 ± 5.00 pC, n = 22) and asynchronous charge (WT 22 °C: 14.02 ± 2.41 pC; WT 32 °C: 10.40 ± 5.16 pC, n = 17; 9Pro 22 °C: 19.83 ± 12.6 pC; 9Pro 32 °C: 14.17 ± 4.42 pC, n = 22) separately. (**j**) Boxplot with fraction of late-train charge transfer during the final 20 pulses released synchronously (WT 22 °C: 0.05344 ± 0.00995, n = 19; 9Pro 22 °C: 0.07128 ± 0.0256, n = 22; WT 32 °C: 0.5444 ± 0.1122, n = 17; 9Pro 32 °C: 0.3707 ± 0.0648, n = 22). (**k**,**l**) Typical traces of asynchronous tail release, normalized to peak release at 22 °C and 32 °C respectively for Syt1 WT and 9Pro expressing synapses. (**m**) Boxplot with decay tau from single exponential fits of asynchronous tail release in Syt1 WT and 9Pro neurons at 22 °C and 32 °C (WT 22 °C: τ = 0.4956 ± 0.127 s, n = 19; 9Pro 22 °C: τ = 0.6869 ± 0.160 s, n = 21; WT 32 °C: τ = 0.1534 ± 0.0879 s, n = 16; 9Pro 32 °C: τ = 0.1762 ± 0.0743 s, n = 21). (*p < 0.05, Wilcoxon signed-rank test for paired and Wilcoxon rank sum test for independent samples).

In order to determine the role of Syt1 mediated suppression of asynchronous release in the temperature dependent change in late-train synchronous release, we recorded hippocampal Syt1 KO autapses expressing Syt1 WT or 9Pro at 22 °C and 32 °C. Basal release parameters in both Syt1 WT and 9Pro expressing synapses responded similarly to temperature shifts. Both showed a 4-fold lower mEPSC frequency at 22 °C, while frequencies overall were higher for Syt1 9Pro (Supplementary Fig. S8a,b). We observed no significant effects on first evoked responses (Supplementary Fig. S8c,d). At RT, during 10 Hz and 20 Hz stimulation Syt1 9Pro expressing synapses showed a clear defect in maintaining synchronous release compared to WT (Figs 6j and 7f). At 40 Hz, both Syt1 WT and 9Pro expressing synapses almost completely desynchronized (Fig. 8a,b,f) and similar late-train synchronous fractions were observed at 22 °C (Fig. 8j). However, at 32 °C, Syt1 9Pro expressing synapses did not synchronize release to the same extent as Syt1 WT, and asynchronous release remained dominant (Fig. 8a,c,g). Although both Syt1 WT and 9Pro expressing synapses showed clear temperature-dependent increases in late-train synchronous release (Fig. 8e,i), the synchronous fraction of late-train release in Syt1 9pro expressing synapses was significantly lower than Syt1 WT at 32 °C (Fig. 8j). Finally, after stimulation ended, the decay of asynchronous tail release at 22 °C was slower (Fig. 8k,m), but not at 32 °C (Fig. 8l,m). This is in line with our previous findings (Supplementary Fig. S6), and supports faster Ca^2+^ clearance at 32 °C^14,21^. We conclude that the Syt1 9Pro mutations inhibit temperature dependent synchronization. Impaired release inhibition in Syt1 9Pro expressing synapses likely leads to premature depletion of vesicles by asynchronous release. This leaves insufficient releasable vesicles for late-train synchronous release. We propose that suppression of asynchronous release by Syt1 constitutes a temperature sensitive mechanism for synchronization of release during high-frequency stimulation, and enables frequency coding.

## Discussion

Synchronization of vesicle release to AP firing is a prerequisite for frequency coding at the synapse. We show that lowering the temperature or reducing the inhibitory function of Syt1 by rendering the C2-domain linker region inflexible, impairs synchronous release at high-frequencies. At the same time, asynchronous release is enhanced, in line with the notion that both modes of release compete^22,42^. Based on these findings we propose that the main role of Syt1’s release inhibitory function is to synchronize synaptic transmission by suppressing asynchronous release in between APs in a highly temperature sensitive manner. This novel role of the inhibitory function of Syt1 is independent of its role in synchronizing the first evoked response and contributes together with its known vesicle recruitment and triggering functions to synchronous transmission over a broad frequency bandwidth.

Our data confirmed previously observed differences in the temperature sensitivity of synchronous release in response to a single AP and during high-frequency stimulation in hippocampal synapses^14^. While the first evoked response remained synchronous at 22 °C, with unchanged amplitude and increased width, late-train synchronous release was almost completely abolished (Fig. 2e–g). Based on previous reports, this difference between initial and late-train release cannot be explained by post-synaptic receptor desensitization at RT^15^, or failure of action potential propagation, which has been shown to be extremely reliable at high-frequencies and different temperatures^56–58^. Furthermore, no evidence of impaired action potential propagation at RT is present when release is synchronized through incubation with EGTA-AM (Supplementary Fig. S4).

In the Calyx of Held, late-train release can remain synchronous at RT during frequencies beyond 100 Hz, and both first evoked and late-train EPSCs increase in amplitude and decrease in width after raising temperature^13,15^. An increase in EPSC width is most likely caused primarily by presynaptic processes, such as broadening of the Ca^2+^ peak^59,60^ due to an increased AP width^15^ and reduced buffering and extrusion of Ca^2+^ at 22 °C^14,21^. The fact that at the same time late-train synchronous release is reduced at 22 °C suggests that an additional mechanism is at work to synchronize release during high-frequency stimulation, which becomes less efficient at lower temperatures. In the Calyx this process might be more efficient at RT or these synapses may exploit other principles to synchronize release during trains.

Temperature dependent changes in calcium currents^13,15,20^, and/or calcium clearance^14,21^ have been proposed as possible mechanisms for temperature sensitive synchronization of late-train release. Broader AP induced calcium peaks and slower calcium buffering/extrusion may increase global intracellular Ca^2+^ at 22 °C, thus promoting asynchronous release^43^. Consequently, given competition between synchronous and asynchronous release for the same vesicle pool^22,42^, synchronous release would be reduced. Indeed, we found that both buffering global intracellular Ca^2+^ with EGTA-AM and raising temperature achieved similar increases in late-train synchronous release (Supplementary Fig. S4; Fig. 2). However, the two conditions differed in that increasing temperature increased total release whereas EGTA-AM lowered it. Presumably lower levels of global intracellular Ca^2+^ reduced refiling of the vesicle pool in the latter case^47–50^. Furthermore, in Syt1 KD synapses, where release depends entirely on global Ca^2+^ ^38^, raising the temperature did not suppress but instead increased asynchronous release during stimulation (Fig. 3). At the same time, Syt1 KD synapses showed faster decay of asynchronous tail release after stimulation, indicative of faster Ca^2+^ clearance at 32 °C. These observations suggest that during high-frequency stimulation, Ca^2+^ influx outcompetes Ca^2+^ clearance and global intracellular Ca^2+^ reaches levels high enough to support the increase in total release we observed in Syt1 KD synapses at 32 °C. Therefore, we conclude that faster Ca^2+^ dynamics are not the dominant factor in temperature dependent synchronization in hippocampal synapses.

In addition to triggering synchronous release^23,24^ Syt1 suppresses asynchronous release^37–39,54,55^. This function is evident when Syt1’s fusion triggering function is selectively impaired in the D363N mutant with reduced Ca^2+^-binding to the C2B domain, displaying that inhibition persist even during high-frequency stimulation (Fig. 4). Suppression of asynchronous release by Syt1 could provide a competitive advantage to synchronous release, and thereby represent the mechanism for temperature-dependent synchronization. Until now, studies of Syt1’s inhibitory function have focussed on spontaneous release^39,54,55^ and suggested a role, either in maintaining the synaptic vesicle pool by preventing “leakage” of vesicles^26,61^, or in suppressing biological noise^12^. In line with these reports, we confirmed that impaired inhibition in the Syt1 9Pro mutant leads to increased spontaneous release^54,55^. However, this did not cause a defect in the first evoked response or total release during high-frequency stimulation (Fig. 6). As such, a role for the inhibitory function in maintaining the synaptic vesicle pool is unlikely. Interestingly, during high-frequency stimulation, Syt1 9Pro expressing synapses showed faster depression of synchronous release, while, concomitantly, asynchronous release increased (Figs 6–8), and persisted for longer (Supplementary Fig. S6). In accordance with a competition model, the defect in synchronization could be completely rescued by suppressing asynchronous release with EGTA-AM (Fig. 7), bypassing the inhibitory role of Syt1, and excluding a possible defect in Ca^2+^-dependent release triggering due to the 9Pro mutations. However, rescue was only partial when raising recording temperature from 22 °C to 32 °C (Fig. 8). As we showed global Ca^2+^ persists at 32 °C (Fig. 3), and the 9Pro mutations impair temperature dependent synchronization, it is likely that raising temperature requires Syt1’s release inhibitory function to synchronize release. In contrast, spontaneous release increased with temperature, regardless of the 9Pro mutations (Supplementary Fig. S8a,b). This may indicate a basal increase in fusogenicity, independent of Syt1’s suppression of asynchronous release. Overall, we propose that preventing asynchronous release during stimulation is the main purpose of Syt1’s release inhibitory function. While suppression of spontaneous release undoubtedly has relevance, it is likely secondary to the increased maintenance of synchronous release achieved by inhibition of the asynchronous component, which directly affects the neurons signalling capabilities.

Syt1 mediated vesicle recruitment, or plasma membrane attachment, has also been shown to aid in synchronization of release, both in a constitutive and Ca^2+^-dependent manner^28^. This notion fits within a proposed sequential two-step release model, where a pre-release docking step separates synchronous release from asynchronous release^62–64^. The same residues required for constitutive recruitment^28^ are targeted in the Syt1 3K mutant used in this study. While we originally wished to employ the Syt1 3K mutant to test to role of Syt1’s inhibitory function in temperature dependent synchronization of release, we did not find any evidence of impaired release inhibition due to these mutations, in contrast to previous results in the Calyx of Held^39^. We did observe a reduced first evoked response in Syt1 3K compared to Syt1 WT expressing synapses (Fig. 5), in line with a role in recruitment. The defect in synchronous release after a single AP was rescued during high-frequency stimulation (Fig. 5). This suggests that Ca^2+^-dependent vesicle recruitment remains intact in Syt1 3K expressing synapses and contributes to late-train synchronous release during high-frequency activity. However, accelerated vesicle recruitment alone is not sufficient to maintain synchronous release during high-frequency stimulation. Firstly, vesicles recruited during high-frequency stimulation tend to release asynchronously due to global intracellular Ca^2+^ ^22,42,43^. Secondly, reducing Ca^2+^-dependent vesicle recruitment by buffering global intracellular Ca^2+^ actually leads to an increase in synchronous charge in WT synapses (Supplementary Fig. S4)^22,42^, instead of a reduction.

**Figure 9:**
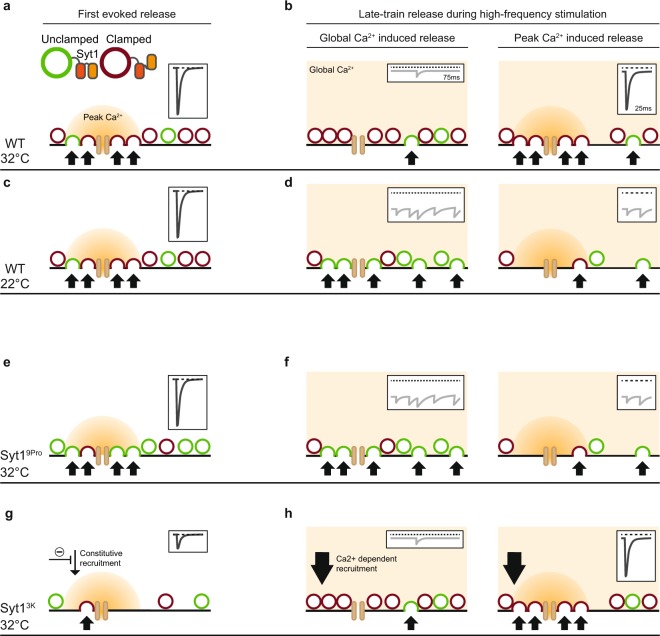
Syt1’s release inhibitory function synchronizes release during high-frequency stimulation. Working model for the effect of Syt1’s release inhibitory and vesicle recruitment functions on release coming from rest (First evoked release; left column) and during high-frequency stimulation (right column), split between global Ca^2+^ induced release in the interpulse-interval (i.e. 75 ms), and peak Ca^2+^ induced release right after the AP (i.e. 25 ms). With vesicles not subject to Syt1’s release inhibitory function in green (unclamped), and clamped vesicles in red. (**a**,**c**,**e**,**g**) First evoked release after a period of rest during peak Ca^2+^ in WT synapses at 32 °C (**a**), in WT synapses at 22 °C (**c**), in Syt1 9Pro synapses at 32 °C with a shift towards unclamped vesicles due to impaired Syt1 release inhibition (**e**), and in Syt1 3K synapses at 32 °C with a decreased number of available vesicles due to impaired constitutive vesicle recruitment (**g**). (**b**,**d**,**f**,**h**) Late-train release during high-frequency stimulation, due to global Ca^2+^ (left), and peak Ca^2+^ (right), in WT synapses at 32 °C (**b**) where rapid transition of newly primed vesicles to the clamped (red) state suppresses asynchronous release due to global Ca^2+^ (**b**, left), preserving vesicles for synchronous release during peak Ca^2+^ (**b**, right). In WT synapses at 22 °C (**d**), where a slower on-rate of release inhibition leaves vesicles unclamped causing asynchronous release (**d**, left), and leaving insufficient vesicles for a synchronous response (**d**, right). In Syt1 9Pro synapses at 32 °C (**f**) where impaired release inhibition leaves vesicles unclamped (**f**, left), leaving too few vesicles for synchronous release (**f**, right). And in Syt1 3 K synapses at 32 °C (**h**) where global Ca^2+^ drives increased Ca^2+^-dependent recruitment, increasing the available pool for synchronous release (**h**, right). In all panels insets show representations of the post-synaptic response based on the depicted fusion events, with synchronous responses in dark grey, asynchronous responses in light grey, and the baseline represented by the dashed line.

Instead, we propose a three-step mechanism by which Syt1 supports synchronous release during low- and high-frequency activity. First, Syt1 recruits vesicles to the plasma membrane to establish or refill the synaptic vesicle pool. Second, Syt1 suppresses global Ca^2+^ triggered asynchronous release. Third, Syt1 triggers synaptic vesicle release upon peak Ca^2+^, coupled to the AP. Under near-physiological conditions (WT; 32 °C), Syt1 aids in providing a sufficient vesicle pool at rest (Fig. 9a), capable of producing a synchronous response upon peak Ca^2+^ after an AP (Fig. 9a; inset). During high-frequency stimulation, asynchronous release is prevented (Fig. 9b left; inset) due to Syt1’s release inhibitory function (clamped vesicles; Fig. 9b left), reserving these vesicles for synchronous release with peak Ca^2+^ upon the next AP (Fig. 9b right). When temperature is lowered (WT; 22 °C), release at rest is not affected (Fig. 9c), however, due to the temperature dependent slowing of the on-rate of Syt1’s inhibitory function, during late-train release, newly primed vesicles are released asynchronously with global Ca^2+^ before they can be clamped (Fig. 9d left). This depletes the available vesicles for the next peak Ca^2+^ (Fig. 9d right), leaving insufficient vesicles for synchronous release (Fig. 9d right; inset). Similarly, when Syt1’s inhibitory function is impaired (Syt1 9Pro), global Ca^2+^ depletes the available vesicles asynchronously (Fig. 9f), and a synchronous peak cannot be generated during the late train (Fig. 9f right; inset). However, Syt1’s inhibitory function does not affect release from a state of rest, where the only Ca^2+^ trigger available is peak Ca^2+^ synchronous with the AP (Fig. 9e). In contrast, impairing Syt1’s constitutive vesicle recruitment function reduces the available pool for synchronous release at rest (Fig. 9g), leading to a decrease in the synchronous response (Fig. 9g; inset). However, during high-frequency stimulation, increases in Ca^2+^-dependent vesicle recruitment driven by global Ca^2+^, provide a strong supply of vesicles (Fig. 9h), supporting synchronous release upon peak Ca^2+^ (Fig. 9h right; inset). In conclusion, we argue that Syt1’s inhibitory function acts independently, but in cooperation with its Ca^2+^-dependent release triggering^23,39^ and vesicle recruitment functions^25,27,28^, to maintain synchronous release and enable frequency coding over a broad frequency bandwidth.

Until now, little is known about the molecular mechanism of release inhibition by Syt1. Recent structural studies have exposed two separate interfaces of the Syt1 C2B domain with the primed SNARE complex^65,66^, both important for fusion. One of these, the tripartite interface, was additionally implicated in establishing a ‘lock’ on release^66^. One possibility is that in the Syt1 9Pro mutation, fixing the orientation of the C2B domains impairs the inhibitory function by preventing optimal interaction with the SNAREs^55^. Increased release inhibition by Syt1 at 32 °C could be due to temperature dependent increases in the association and dissociation constants for the tripartite interface with the SNAREs. This would not affect the steady state of the inhibited, or “clamped”, pool of vesicles (Fig. 9a), but would speed up the clamping of newly primed vesicles after late-train synchronous release of previously clamped vesicles.

Interestingly, several other C2B domain containing proteins can associate with SNAREs at the tripartite interface^66^. Among them, Synaptotagmin-7 (Syt7), which has been implicated in triggering asynchronous release^52,67–69^. This is in line with a model where distinct Ca^2+^-sensors compete for SNARE-binding, thus defining the balance between synchronous and asynchronous release^66,70^. Indeed, a recent study shows that neurons control the relative contributions of synchronous and asynchronous release, and thereby the ability to encode spike trains, by regulating expression of fast Synaptotagmin isoforms^71^. However, other pathways of suppression, such as direct inhibition of a second Ca^2+^ sensor for asynchronous release cannot be excluded^39^.

Despite asynchronous release inhibition by Syt1, several examples of persisting asynchronous release under physiological conditions are known^69,71–75^. In the hippocampus, inhibitory cholecystokinin (CCK) interneurons display substantial asynchronous release after burst of activity, presumably to prolong release inhibition^72^. Similarly, in the cortex, asynchronous GABA release is thought to decrease generalized synchronous firing, preventing epileptic activity^75^. Furthermore, asynchronous release triggered by Syt7 is proposed to help in boosting post-synaptic firing probability during high-frequency stimulation^68^. Interestingly, Syt7 has also been described to contribute to synchronous release as a sensor for short-term plasticity^69,76,77^, and establishing amplitude frequency invariance^77,78^. One way to explain the involvement in these two processes is the ability of Syt7 or other slow Ca^2+^ sensors to reduce the energy barrier for vesicle fusion when activated by global intracellular Ca^2+^. Activation of a slow Ca^2+^ sensor by itself will trigger asynchronous release, thus desynchronizing release. However, when it is activated simultaneously with Syt1, their additive effect on the energy barrier could lead to multiplicative effects on the vesicle fusion rate, requiring only little activation of the slow sensor to boost Syt1 action^77,79,80^. In this scenario, Syt1 performs a balancing act by allowing just enough activation of a second Ca^2+^ sensor to enhance synchronous release during AP’s without inducing substantial premature release of vesicles in between AP’s.

In conclusion, we identified a novel aspect of the inhibitory function of Syt1 in maintaining synchronous transmission over a wide range of frequencies by suppressing asynchronous release. This function appears to be independent of its fusion promoting, and vesicle recruitment functions, and presumably acts by (partially) inhibiting slow Ca^2+^ sensors. All these functions of Syt1 work together to optimize synchronous vesicle release, thus broadening the bandwidth for frequency coded information transfer.

## Materials and Methods

### Animals

Neuronal cultures were prepared from embryonic day 18 (E18) pups of both sexes, obtained by caesarean section of pregnant female mice. For WT neurons, the C57BL/6 mouse line was used (Figs 1–3), and a previously described Synaptotagmin-1 knockout^23^ line was used for Syt1 KO neurons (Figs 4–8). New-born pups (P0-P1) from Winstar rats were used for glia preparations. Animals were housed and bred according to institutional and Dutch governmental guidelines, and all procedures are approved by the ethical committee of the Vrije Universiteit, Amsterdam, The Netherlands (Dierexperimentencomissie (DEC) license number: FGA11-03).

### Dissociated neuronal cultures

Hippocampi from WT and Syt1 KO mice were isolated, collected in ice-cold Hank’s buffered salt solution (HBSS; Sigma) buffered with 1 mM HEPES (Invitrogen), and digested for 20 min with 0.25% trypsin (Invitrogen) at 37 °C. After washing, neurons were dissociated using a fire-polished Pasteur pipette and resuspended in Neurobasal medium supplemented with 2% B-27, 1% HEPES, 0.25% GlutaMAX, and 0.1% Penicillin-Streptomycin (all Invitrogen). Neurons were counted in a Fuchs-Rosenthal chamber and plated at 1.5 K per well in a 12-well plate. Neuronal cultures were maintained in Neurobasal medium supplemented with 2% B-27, 1% HEPES, 0.25% GlutaMAX, and 0.1% Penicillin-Streptomycin (all Invitrogen), at 37 °C in a 5% CO humidified incubator.

Autaptic hippocampal cultures were prepared as described previously^41^. Briefly, micro-islands were prepared with a solution containing 0.1 mg/ml poly-D-lysine (sigma), 0.7 mg/ml rat tail collagen (BD Biosciences) and 10 mM acetic acid (Sigma) applied with a custom-made rubber stamp (dot diameter 250 μm). Next, rat astrocytes were plated at 6–8 K per well in pre-warmed DMEM (Invitrogen), supplemented with 10% FCS, 1% Penicillin-Streptomycin and 1% nonessential amino acids (All Gibco).

### Constructs and lentiviral infection

The Synaptotagmin-1 mutations Syt1 D363N, and Syt1 3 K (K326Q, K327Q, K331Q), were generated using Quickchange (Stratagene) and verified by sequencing, Syt1 9Pro (residues 264–272 replaced with nine proline residues) as previously described^54,55^, and was kindly provided by Dr. Edwin Chapman, (Howard Hughes Medical Institute, Madison, WI, USA). For Syt1 knockdown, we used anti-Syt1 shRNA described previously^52^, kindly provided by Thomas Südhof (Howard Hughes Medical Institute, Stanford, CA, USA). For expression in neurons, constructs were subcloned into synapsin-promotor-driven-pLenti vectors, and viral particles were produced as described^81^. For Syt1 knockdown, WT neurons were infected with viral particles at DIV 2, and for rescue experiments, Syt1 KO neurons were infected at DIV4.

### Electrophysiology

Whole‐cell voltage‐clamp recordings (Vm = −70 mV) were performed on autaptic neurons DIV14-19, with borosilicate glass pipettes (2–5MΩ) filled with (in mM) 125 K+‐gluconic acid, 10 NaCl, 4.6 MgCl2,4K2‐ATP, 15 creatine phosphate, 1 EGTA, and 10 units/mL phosphocreatine kinase (pH 7.30). External solution contained in mM: 10 HEPES, 10 glucose, 140 NaCl, 2.4 KCl, 4 MgCl2, and 4 CaCl2 (pH = 7.30, 300 mOsmol). Inhibitory neurons were identified and excluded based on the decay of postsynaptic currents. Recordings were acquired with a MultiClamp 700B amplifier, Digidata 1440 A, and pCLAMP 10.3 software (Molecular Devices). Only cells with an access resistance < 15MΩ (80% compensated) and leak current of <300 pA were included. EPSCs were elicited by a 0.5 ms depolarization to 30 mV.

In experiments regarding the effect of temperature, cells were controlled at 22 ± 1 °C and 32.5 ± 1 °C using bath perfusion with warm or cold ACSF. For these experiments, bath temperature was monitored continuously using two temperature sensors (TS-100; npi Electronic GmbH), one of which was placed near the bath outflow and provided feedback to an inline solution heater while the other sensor was positioned near the patched cell and used to register the precise temperature during recordings. All other recordings were performed at room temperature (RT) 22 ± 2 °C unmonitored. For experiments including the cell permeable Ca^2+^ chelator EGTA-AM, cells were recorded at RT before or after 10 min incubation with 50 µM EGTA-AM in bath. This concentration was chosen to be sufficiently high to avoid buffer saturation during train stimulation, while keeping suppression of EPSC amplitude to a minimum. RRP size was assessed by hypertonic sucrose application (500 mM for 7 s)^79,82^, using a piezo-controlled barrel application system (Perfusion Fast-Step, Warner Instruments) and fitted as described previously^79^.

### Data analysis

Offline analysis was performed using custom‐written software routines in Matlab R2018b (Mathworks). In all figures, stimulation artefacts have been removed. For evoked release, total charge was calculated by integrating the current from the end of the stimulation until the start of the next pulse. Synchronous release was separated from asynchronous release by subtracting the standing current at the start of the EPSC and integrating the first 25 ms of the stimulation, referred to as the synchronous window (Supplementary Fig. S2). This window was chosen based on a comparison of release per pulse before and after incubation with EGTA-AM, under the assumption that most asynchronous release is blocked by buffering global Ca^2+^, and provided an objective estimate of the synchronous component (see main text). Asynchronous release per stimulation interval was determined by subtracting the synchronous charge from the total charge. Statistical significance was determined using Wilcoxon signed-rank tests and Mann-Whitney *U* tests to compare paired- and independent measurements, respectively, p-values below 0.05 were considered significant. All statistical tests were performed in Matlab (Mathworks).

## Supplementary information

Supplementary Figures

## References

[1] deCharms RC, Zador A (2000). Neural representation and the cortical code. Annu. Rev. Neurosci..

[2] Delvendahl I, Hallermann S (2016). The Cerebellar Mossy Fiber Synapse as a Model for High-Frequency Transmission in the Mammalian CNS. Trends Neurosci..

[3] Izhikevich EM, Desai NS, Walcott EC, Hoppensteadt FC (2003). Bursts as a unit of neural information: selective communication via resonance. Trends Neurosci..

[4] London M, Roth A, Beeren L, Häusser M, Latham PE (2010). Sensitivity to perturbations *in vivo* implies high noise and suggests rate coding in cortex. Nature.

[5] O’Keefe J, Dostrovsky J (1971). The hippocampus as a spatial map. Preliminary evidence from unit activity in the freely-moving rat. Brain Res..

[6] Pastalkova E, Itskov V, Amarasingham A, Buzsáki G (2008). Internally generated cell assembly sequences in the rat hippocampus. Science.

[7] MacDonald CJ, Lepage KQ, Eden UT, Eichenbaum H (2011). Hippocampal ‘time cells’ bridge the gap in memory for discontiguous events. Neuron.

[8] Wood ER, Dudchenko PA, Eichenbaum H (1999). The global record of memory in hippocampal neuronal activity. Nature.

[9] Terada S, Sakurai Y, Nakahara H, Fujisawa S (2017). Temporal and Rate Coding for Discrete Event Sequences in the Hippocampus. Neuron.

[10] Lorteije JAM, Rusu SI, Kushmerick C, Borst JGG (2009). Reliability and precision of the mouse calyx of Held synapse. J. Neurosci..

[11] McNaughton BL, Barnes CA, O’Keefe J (1983). The contributions of position, direction, and velocity to single unit activity in the hippocampus of freely-moving rats. Exp. brain Res..

[12] Südhof TC (2013). Neurotransmitter release: the last millisecond in the life of a synaptic vesicle. Neuron.

[13] Taschenberger H, von Gersdorff H (2000). Fine-tuning an auditory synapse for speed and fidelity: developmental changes in presynaptic waveform, EPSC kinetics, and synaptic plasticity. J. Neurosci..

[14] Pyott SJ, Rosenmund C (2002). The effects of temperature on vesicular supply and release in autaptic cultures of rat and mouse hippocampal neurons. J. Physiol..

[15] Kushmerick C, Renden R, von Gersdorff H (2006). Physiological temperatures reduce the rate of vesicle pool depletion and short-term depression via an acceleration of vesicle recruitment. J. Neurosci..

[16] Maximov A, Pang ZP, Tervo DGR, Südhof TC (2007). Monitoring synaptic transmission in primary neuronal cultures using local extracellular stimulation. J. Neurosci. Methods.

[17] Fernández-Alfonso T, Ryan TA (2004). The kinetics of synaptic vesicle pool depletion at CNS synaptic terminals. Neuron.

[18] Micheva KD, Smith SJ (2005). Strong effects of subphysiological temperature on the function and plasticity of mammalian presynaptic terminals. J. Neurosci..

[19] Volgushev M (2004). Probability of transmitter release at neocortical synapses at different temperatures. J. Neurophysiol..

[20] Nouvian R (2007). Temperature enhances exocytosis efficiency at the mouse inner hair cell ribbon synapse. J. Physiol..

[21] Klyachko VA, Stevens CF (2006). Temperature-dependent shift of balance among the components of short-term plasticity in hippocampal synapses. J. Neurosci..

[22] Otsu Y (2004). Competition between phasic and asynchronous release for recovered synaptic vesicles at developing hippocampal autaptic synapses. J. Neurosci..

[23] Geppert M (1994). Synaptotagmin I: a major Ca2+ sensor for transmitter release at a central synapse. Cell.

[24] Nishiki T, Augustine GJ (2004). Synaptotagmin I synchronizes transmitter release in mouse hippocampal neurons. J. Neurosci..

[25] de Wit H (2009). Synaptotagmin-1 docks secretory vesicles to syntaxin-1/SNAP-25 acceptor complexes. Cell.

[26] Imig C (2014). The morphological and molecular nature of synaptic vesicle priming at presynaptic active zones. Neuron.

[27] Kedar GH (2015). A Post-Docking Role of Synaptotagmin 1-C2B Domain Bottom Residues R398/399 in Mouse Chromaffin Cells. J. Neurosci..

[28] Chang Shuwen, Trimbuch Thorsten, Rosenmund Christian (2017). Synaptotagmin-1 drives synchronous Ca2+-triggered fusion by C2B-domain-mediated synaptic-vesicle-membrane attachment. Nature Neuroscience.

[29] Nicholson-Tomishima K, Ryan TA (2004). Kinetic efficiency of endocytosis at mammalian CNS synapses requires synaptotagmin I. Proc. Natl. Acad. Sci..

[30] Poskanzer KE, Marek KW, Sweeney ST, Davis GW (2003). Synaptotagmin I is necessary for compensatory synaptic vesicle endocytosis *in vivo*. Nature.

[31] Poskanzer KE, Fetter RD, Davis GW (2006). Discrete Residues in the C2B Domain of Synaptotagmin I Independently Specify Endocytic Rate and Synaptic Vesicle Size. Neuron.

[32] Yao L-H (2012). Synaptotagmin 1 Is Necessary for the Ca2+ Dependence of Clathrin-Mediated Endocytosis. J. Neurosci..

[33] Li YC, Chanaday NL, Xu W, Kavalali ET (2017). Synaptotagmin-1- and Synaptotagmin-7-Dependent Fusion Mechanisms Target Synaptic Vesicles to Kinetically Distinct Endocytic Pathways. Neuron.

[34] Broadie K, Bellen HJ, DiAntonio A, Littleton JT, Schwarz TL (1994). Absence of synaptotagmin disrupts excitation-secretion coupling during synaptic transmission. Proc. Natl. Acad. Sci. USA.

[35] Chicka MC, Hui E, Liu H, Chapman ER (2008). Synaptotagmin arrests the SNARE complex before triggering fast, efficient membrane fusion in response to Ca2. Nat. Struct. Mol. Biol..

[36] Littleton JT, Stern M, Perin M, Bellen HJ (1994). Calcium dependence of neurotransmitter release and rate of spontaneous vesicle fusions are altered in Drosophila synaptotagmin mutants. Proc. Natl. Acad. Sci. USA.

[37] Nishiki T, Augustine GJ (2004). Dual roles of the C2B domain of synaptotagmin I in synchronizing Ca^2+^-dependent neurotransmitter release. J. Neurosci..

[38] Maximov A, Südhof TC (2005). Autonomous function of synaptotagmin 1 in triggering synchronous release independent of asynchronous release. Neuron.

[39] Kochubey O, Schneggenburger R (2011). Synaptotagmin increases the dynamic range of synapses by driving Ca^2+^-evoked release and by clamping a near-linear remaining Ca^2+^ sensor. Neuron.

[40] Sun J (2007). A dual-Ca2+-sensor model for neurotransmitter release in a central synapse. Nature.

[41] Meijer M (2018). Tyrosine phosphorylation of Munc18-1 inhibits synaptic transmission by preventing SNARE assembly. EMBO J..

[42] Hagler DJ, Goda Y (2001). Properties of synchronous and asynchronous release during pulse train depression in cultured hippocampal neurons. J. Neurophysiol..

[43] Atluri PP, Regehr WG (1998). Delayed Release of Neurotransmitter from Cerebellar Granule Cells. J. Neurosci..

[44] Adler EM, Augustine GJ, Duffy SN, Charlton MP (1991). Alien intracellular calcium chelators attenuate neurotransmitter release at the squid giant synapse. J. Neurosci..

[45] Winslow JL, Duffy SN, Charlton MP (1994). Homosynaptic facilitation of transmitter release in crayfish is not affected by mobile calcium chelators: implications for the residual ionized calcium hypothesis from electrophysiological and computational analyses. J. Neurophysiol..

[46] Feller MB, Delaney KR, Tank DW (1996). Presynaptic calcium dynamics at the frog retinotectal synapse. J. Neurophysiol..

[47] Dittman JS, Regehr WG (1996). Contributions of calcium-dependent and calcium-independent mechanisms to presynaptic inhibition at a cerebellar synapse. J. Neurosci..

[48] Wang LY, Kaczmarek LK (1998). High-frequency firing helps replenish the readily releasable pool of synaptic vesicles. Nature.

[49] Hosoi N, Sakaba T, Neher E (2007). Quantitative analysis of calcium-dependent vesicle recruitment and its functional role at the calyx of Held synapse. J. Neurosci..

[50] Sakaba T (2008). Two Ca(2+)-dependent steps controlling synaptic vesicle fusion and replenishment at the cerebellar basket cell terminal. Neuron.

[51] Yoshihara M, Guan Z, Littleton JT (2010). Differential regulation of synchronous versus asynchronous neurotransmitter release by the C2 domains of synaptotagmin 1. Proc. Natl. Acad. Sci. USA.

[52] Bacaj T (2013). Synaptotagmin-1 and synaptotagmin-7 trigger synchronous and asynchronous phases of neurotransmitter release. Neuron.

[53] Xu J, Pang ZP, Shin O-H, Südhof TC (2009). Synaptotagmin-1 functions as a Ca^2+^ sensor for spontaneous release. Nat. Neurosci..

[54] Liu H (2014). Linker mutations reveal the complexity of synaptotagmin 1 action during synaptic transmission. Nat. Neurosci..

[55] Bai H (2016). Different states of synaptotagmin regulate evoked versus spontaneous release. Nat. Commun..

[56] Cox CL, Denk W, Tank DW, Svoboda K (2000). Action potentials reliably invade axonal arbors of rat neocortical neurons. Proc. Natl. Acad. Sci..

[57] Raastad M, Shepherd GMG (2003). Single-axon action potentials in the rat hippocampal cortex. J. Physiol..

[58] Radivojevic, M. *et al*. Tracking individual action potentials throughout mammalian axonal arbors. *Elife* **6** (2017). 10.7554/eLife.30198 PMC5633342 28990925

[59] Borst JG, Sakmann B (1998). Calcium current during a single action potential in a large presynaptic terminal of the rat brainstem. J. Physiol..

[60] Borst JG, Helmchen F (1998). Calcium influx during an action potential. Methods Enzymol..

[61] Morimoto T, Wang X, Poo M (1997). Overexpression of synaptotagmin modulates short-term synaptic plasticity at developing neuromuscular junctions. Neuroscience.

[62] Miki T (2016). Actin- and Myosin-Dependent Vesicle Loading of Presynaptic Docking Sites Prior to Exocytosis. Neuron.

[63] Miki T, Nakamura Y, Malagon G, Neher E, Marty A (2018). Two-component latency distributions indicate two-step vesicular release at simple glutamatergic synapses. Nat. Commun..

[64] Neher E, Brose N (2018). Dynamically Primed Synaptic Vesicle States: Key to Understand Synaptic Short-Term Plasticity. Neuron.

[65] Zhou Q (2015). Architecture of the synaptotagmin-SNARE machinery for neuronal exocytosis. Nature.

[66] Zhou Qiangjun, Zhou Peng, Wang Austin L., Wu Dick, Zhao Minglei, Südhof Thomas C., Brunger Axel T. (2017). The primed SNARE–complexin–synaptotagmin complex for neuronal exocytosis. Nature.

[67] Luo F, Bacaj T, Südhof TC (2015). Synaptotagmin-7 Is Essential for Ca2+-Triggered Delayed Asynchronous Release But Not for Ca2+-Dependent Vesicle Priming in Retinal Ribbon Synapses. J. Neurosci..

[68] Luo F, Südhof TC (2017). Synaptotagmin-7-Mediated Asynchronous Release Boosts High-Fidelity Synchronous Transmission at a Central Synapse. Neuron.

[69] Turecek J, Regehr WG (2018). Synaptotagmin 7 Mediates Both Facilitation and Asynchronous Release at Granule Cell Synapses. J. Neurosci..

[70] Walter AM, Groffen AJ, Sørensen JB, Verhage M (2011). Multiple Ca2+ sensors in secretion: teammates, competitors or autocrats?. Trends Neurosci..

[71] Turecek Josef, Regehr Wade G. (2019). Neuronal Regulation of Fast Synaptotagmin Isoforms Controls the Relative Contributions of Synchronous and Asynchronous Release. Neuron.

[72] Hefft S, Jonas P (2005). Asynchronous GABA release generates long-lasting inhibition at a hippocampal interneuron-principal neuron synapse. Nat. Neurosci..

[73] Iremonger KJ, Bains JS (2007). Integration of asynchronously released quanta prolongs the postsynaptic spike window. J. Neurosci..

[74] Best AR, Regehr WG (2009). Inhibitory regulation of electrically coupled neurons in the inferior olive is mediated by asynchronous release of GABA. Neuron.

[75] Manseau Frédéric, Marinelli Silvia, Méndez Pablo, Schwaller Beat, Prince David A., Huguenard John R., Bacci Alberto (2010). Desynchronization of Neocortical Networks by Asynchronous Release of GABA at Autaptic and Synaptic Contacts from Fast-Spiking Interneurons. PLoS Biology.

[76] Jackman SL, Turecek J, Belinsky JE, Regehr WG (2016). The calcium sensor synaptotagmin 7 is required for synaptic facilitation. Nature.

[77] Chen C, Satterfield R, Young SM, Jonas P (2017). Triple Function of Synaptotagmin 7 Ensures Efficiency of High-Frequency Transmission at Central GABAergic Synapses. Cell Rep..

[78] Turecek J, Jackman SL, Regehr WG (2017). Synaptotagmin 7 confers frequency invariance onto specialized depressing synapses. Nature.

[79] Schotten S (2015). Additive effects on the energy barrier for synaptic vesicle fusion cause supralinear effects on the vesicle fusion rate. Elife.

[80] Jackman SL, Regehr WG (2017). The Mechanisms and Functions of Synaptic Facilitation. Neuron.

[81] Naldini L (1996). *In vivo* gene delivery and stable transduction of nondividing cells by a lentiviral vector. Science.

[82] Rosenmund C, Stevens CF (1996). Definition of the readily releasable pool of vesicles at hippocampal synapses. Neuron.

