# Publisher Correction: Synaptotagmin-1 enables frequency coding by suppressing asynchronous release in a temperature dependent manner

**DOI:** 10.1038/s41598-019-54233-8

**Published:** 2019-11-22

**Authors:** Vincent Huson, Maaike A. van Boven, Alexia Stuefer, Matthijs Verhage, L. Niels Cornelisse

**Affiliations:** 1 Department of Functional Genomics, Clinical Genetics, Center for Neurogenomics and Cognitive Research, Amsterdam University Medical Center- Location VUmc, Amsterdam, The Netherlands; 2 Department of Functional Genomics, Center for Neurogenomics and Cognitive Research, VU University Amsterdam, Amsterdam, The Netherlands

Correction to: *Scientific Reports* 10.1038/s41598-019-47487-9, published online 05 August 2019

The original version of this Article contained an error in Affiliation 1, which was incorrectly given as ‘Department of Functional Genomics, Clinical Genetics, Center for Neurogenomics and Cognitive Research, University Medical Center Amsterdam, Amsterdam, The Netherlands’. The correct affiliation is listed below:

Department of Functional Genomics, Clinical Genetics, Center for Neurogenomics and Cognitive Research, Amsterdam University Medical Center- Location VUmc, Amsterdam, The Netherlands.

This error has now been corrected in the HTML and PDF versions of the Article, and in the accompanying Supplementary Material.

