# Supplementary material for: Synaptotagmin-1 enables frequency coding by suppressing asynchronous release in a temperature dependent manner
Source: Sci Rep. 2019 Aug 5;9:11341. doi: 10.1038/s41598-019-47487-9 (PMC6683208; doi:10.1038/s41598-019-47487-9)
Supplement: Supplementary file 1 — Supplementary Figures [file 41598_2019_47487_MOESM1_ESM.pdf]

Synaptotagmin-1 enables frequency coding by suppressing asynchronous release in a temperature dependent manner.

Vincent Huson[1†], Maaike van Boven[2†], Alexia Stuefer[2], Matthijs Verhage[1,2], L. Niels Cornelisse[1*].

[1]Department of Functional Genomics, Clinical Genetics, Center for Neurogenomics and Cognitive Research, Amsterdam University Medical Center- Location VUmc, Amsterdam, The Netherlands

[2]Department of Functional Genomics, Center for Neurogenomics and Cognitive Research, VU University Amsterdam, Amsterdam, The Netherlands

[†]These authors contributed equally.

[*]Corresponding author: l.n.cornelisse@vu.nl

Supplementary Figure S1

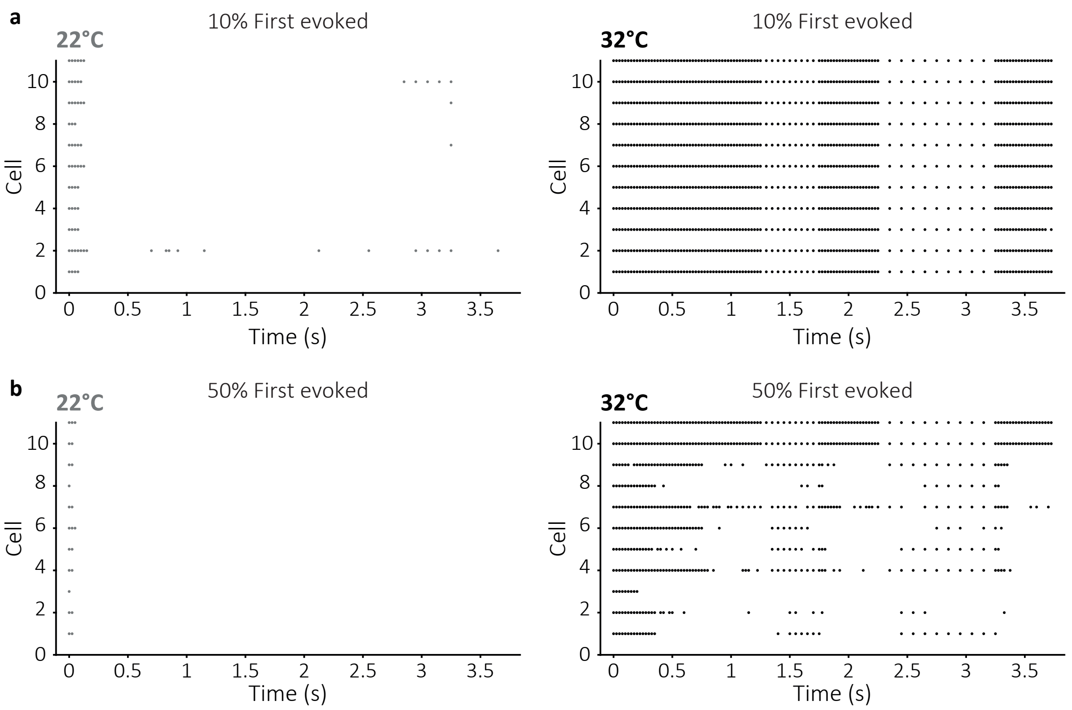

Supplementary Figure S1. Temperature effect on frequency-coded signalling is robust for threshold height

(a) Raster plot of cells firing above 10%, and **(b)** 50% amplitude of first evoked response at 22°C (left) and 32°C (right).

Supplementary Figure S2

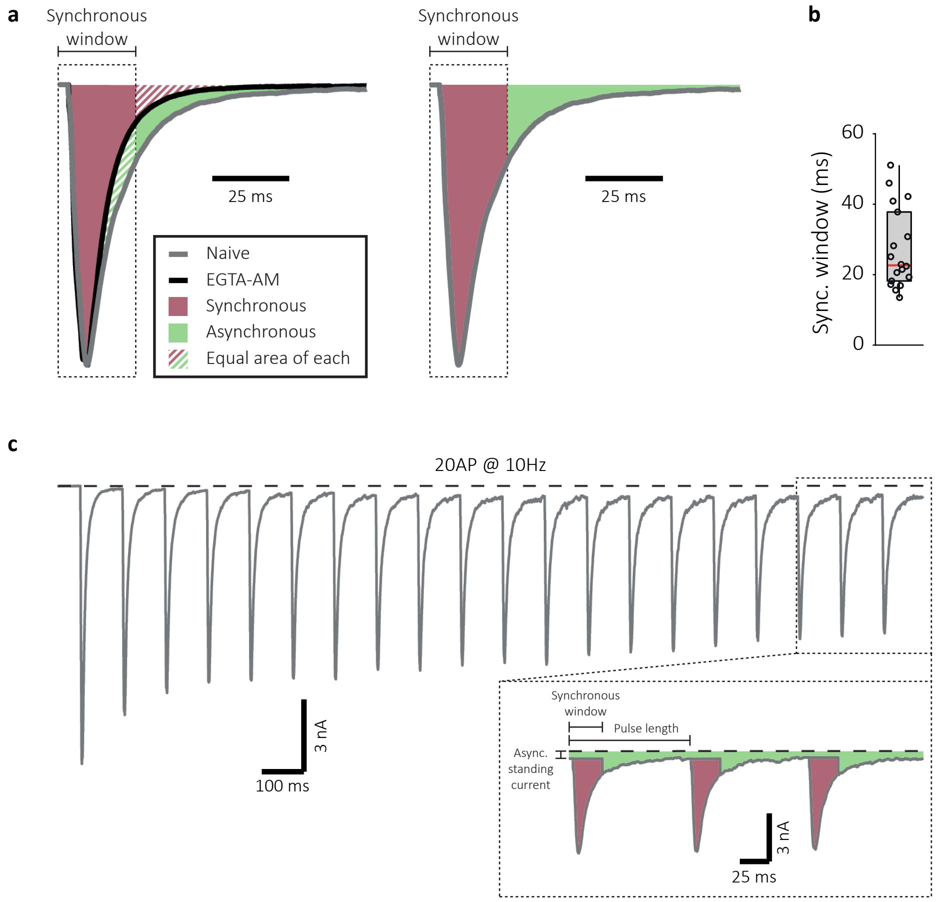

Supplementary Figure S2. Synchronous charge is well estimated by the first 25ms of release

(a) Superimposed traces before (light) and after application of EGTA-AM (dark), scaled to peak amplitude (left); and naive trace showing synchronous (dark) and asynchronous (light) release divided by the point where Naive charge exceeds total EGTA-AM charge for the scaled responses (right). **(b)** Boxplot of synchronous window, showing the point where naive charge exceeds scaled EGTA-AM charge (22.65 ± 5.65ms, n=18). All recordings at room temperature, unmonitored.

Supplementary Figure S3

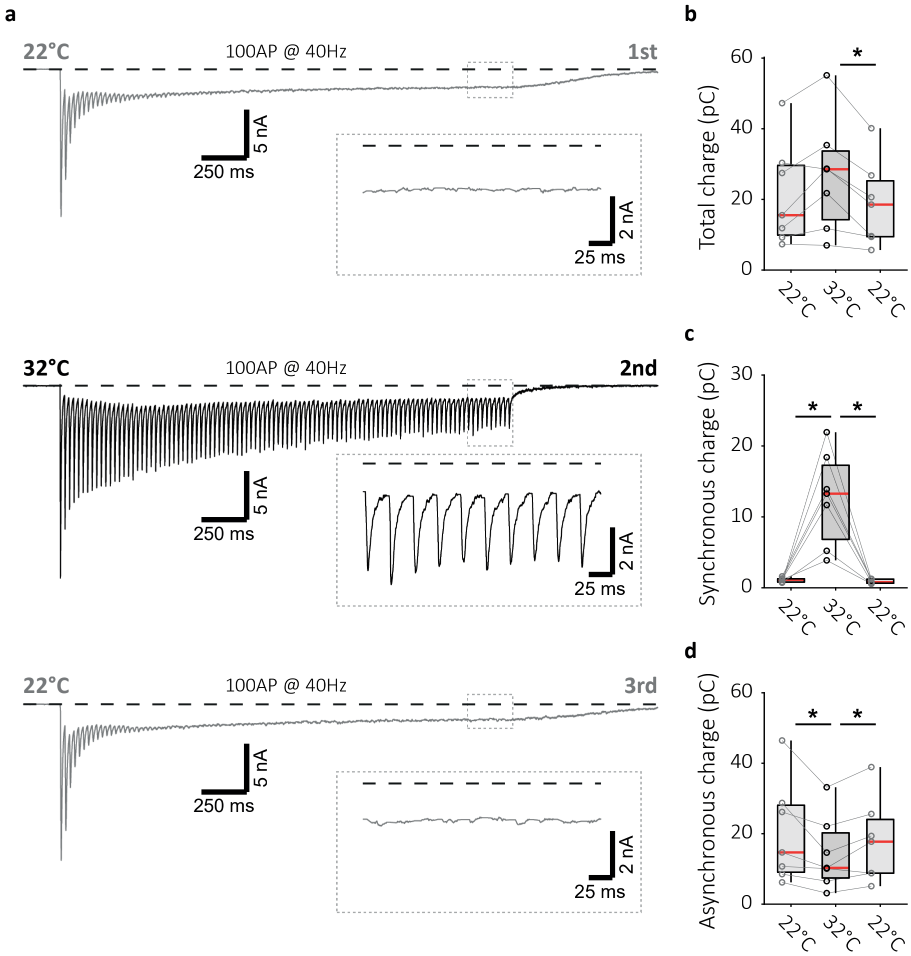

Supplementary Figure S3. Temperature effects on synchronization are completely reversible

(a) Typical traces during 100 pulses at 40Hz stimulation from a cell recorded at 22°C (top; 1st), heated to 32°C (middle; 2nd), and cooled back down to 22°C (bottom; 3rd). **(b)** Boxplots showing late-train charge transferred averaged over the final 20 pulses of the 40Hz train before, during, and after heating to 32°C for **(b)** total charge (22°C 1st: 15.53 ± 8.21 pC; 32°C: 28.54 ± 6.83 pC; 22°C 2nd: 18.53 ± 8.29 pC; n=7), **(c)** synchronous charge (22°C 1st: 1.135 ± 0.293 pC; 32°C: 13.27 ± 5.13 pC; 22°C 2nd: 0.7991 ± 0.239 pC; n=7), and **(d)** asynchronous charge (22°C 1st: 14.69 ± 10.3 pC; 32°C: 10.29 ± 4.34 pC; 22°C 2nd: 17.74 ± 8.93 pC; n=7). (* $p < 0.05$, Wilcoxon signed-rank test).

Supplementary Figure S4

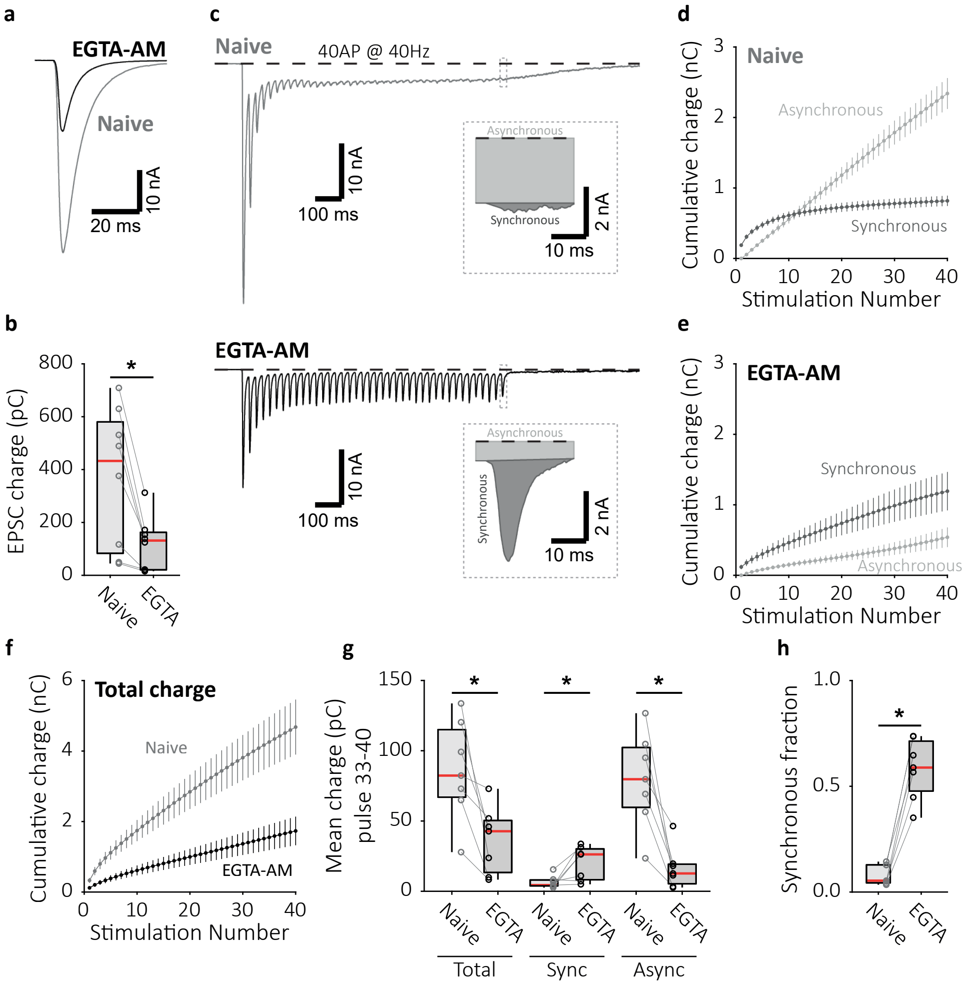

Supplementary Figure S4. Buffering global Ca^{2+} synchronises release but suppresses total charge transfer

(a) Single EPSC representative traces and **(b)** boxplot of charge transferred per EPSC before (Naive) and after application of EGTA-AM (Naive: 432.7 ± 237 pC, n=8; EGTA-AM: 131.5 ± 74.9 pC, n=8). **(c)** Representative traces of 40 Hz train stimulation (40 pulses, 1 s) before (Naive) and after application of EGTA-AM; single pulse zooms display division synchronous and asynchronous release. **(d)** Cumulative plots (mean ± S.E.M.) of charge transferred synchronously and asynchronously before (Naive), and **(e)** after application of EGTA-AM. **(f)** Cumulative total charge (mean ± S.E.M.) Naive and with EGTA-AM. **(g)** Boxplots with late-train charge averaged over the final 8 pulses of the 40Hz train, before and after application of EGTA-AM. Displayed for total charge (Naive: 82.34 ± 17.4 pC, n=7; EGTA-AM: 42.72 ± 19.0 pC, n=7), and synchronous (Naive: 4.399 ± 1.79 pC, n=7; EGTA-AM: 26.25 ± 7.48 pC, n=7) and asynchronous charge (Naive: 79.73 ± 23.2 pC, n=7; EGTA-AM: 12.69 ± 6.99 pC, n=7) separately. **(h)** Boxplot with fraction of late-train charge transfer during the final 8 pulses released synchronously (Naive: 0.05378 ± 0.0192, n=7; EGTA-AM: 0.5885 ± 0.141, n=7). All recordings at room temperature, unmonitored. (* $p < 0.05$, Wilcoxon signed-rank test).

Supplementary Figure S5

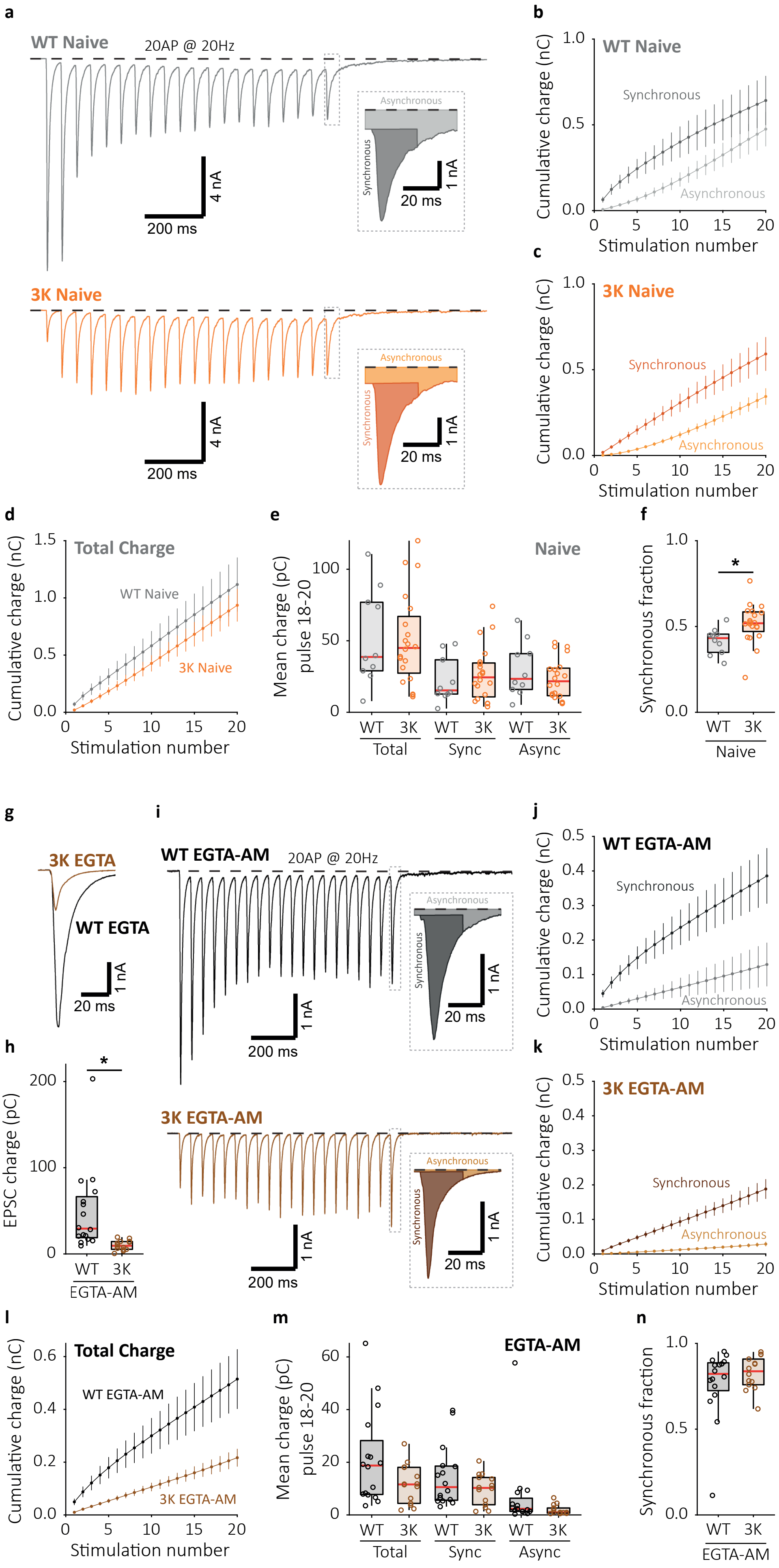

Supplementary Figure S5. Rescues of synchronous release in Syt1 3K neurons depends on global Ca^{2+}

(a) Representative traces of 20 Hz train stimulation (20 pulses, 1 s) in Syt1 WT or 3K expressing synapses in the absence (Naive) or **(i)** presence of EGTA-AM ; single pulse zooms display division synchronous and asynchronous release. **(b)** Cumulative plots (mean ± S.E.M.) of charge transferred synchronously and asynchronously in naive Syt1 WT and **(j)** in the presence of EGTA-AM, or **(c)** naive Syt1 3K expressing synapses and **(k)** in the presence of EGTA-AM. **(d)** Cumulative total charge (mean ± S.E.M.) in naive and **(l)** EGTA-AM Syt1 WT and 3K conditions. **(e)** Boxplots with late-train charge averaged over the final 3 pulses of the 20Hz train, for naive and **(m)** EGTA-AM Syt1 WT and 3K conditions. Displayed for total charge (WT Naive: 38.67 ± 22.0 pC, n=10; 3K Naive: 44.97 ± 19.0 pC, n=20; WT EGTA-AM: 18.75 ± 11.0 pC, n=16; 3K EGTA-AM: 11.59 ± 6.53 pC, n=14), and synchronous (WT Naive: 15.31 ± 24.4 pC, n=10; 3K Naive: 24.42 ± 11.4 pC, n=20; WT EGTA-AM: 10.55 ± 5.35 pC, n=16; 3K EGTA-AM: 10.26 ± 4.95 pC, n=14) and asynchronous charge (WT Naive: 23.36 ± 13.2 pC, n=10; 3K Naive: 21.67 ± 9.83 pC, n=20; WT EGTA-AM: 2.309 ± 1.39 pC, n=16; 3K EGTA-AM: 0.9639 ± 0.415 pC, n=14) separately. **(f,n)** Boxplot with fraction of late-train charge transfer during the final 3 pulses released synchronously for naive and EGTA-AM conditions respectively (WT Naive: 0.4320 ± 0.0384, n=10; 3K Naive: 0.5188 ± 0.0662, n=20; WT EGTA-AM: 0.8223 ± 0.0717, n=16; 3K EGTA-AM: 0.8374 ± 0.07491, n=14). **(g)** Single EPSC representative traces and **(h)** boxplot of charge transferred per EPSC in Syt1 WT and 3K expressing synapses in the presence of EGTA-AM (WT EGTA-AM: 29.39 ± 16.5 pC, n=16; 3K EGTA-AM: 9.480 ± 4.37 pC, n=14). All recordings at room temperature, unmonitored. (* $p < 0.05$, Wilcoxon rank sum test).

Supplementary Figure S6

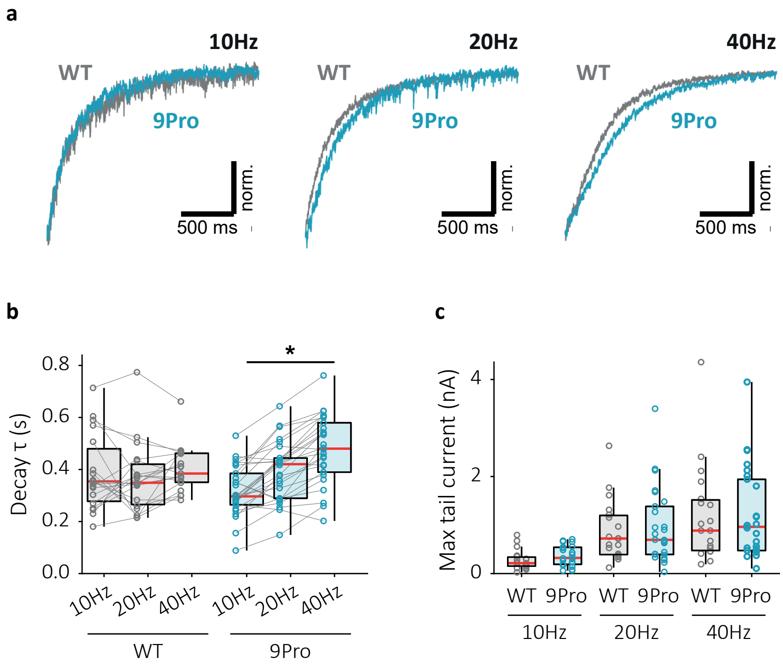

Supplementary Figure S6. Syt1 9Pro mutation increases asynchronous tail release in a frequency dependent manner

(a) Typical traces of asynchronous tail release, normalized to peak release after 10Hz (left), 20Hz (middle), and 40Hz (right) train stimulations, in Syt1 WT and 9Pro expressing synapses. **(b)** Boxplots with decay tau from single exponential fits of asynchronous tail release at 10Hz, 20Hz, and 40Hz, and in Syt1 WT (10Hz: τ=0.3542 ± 0.0850s; 20Hz: τ=0.3489 ± 0.0847s; 40Hz: τ=0.3845 ± 0.0662s, n=19) and 9Pro (10Hz: τ=0.2966 ± 0.0547s; 20Hz: τ=0.4204 ± 0.0800s; 40Hz: τ=0.4800 ± 0.0949s, n=26) expressing synapses. **(c)** Boxplot of maximum asynchronous tail current at 10Hz (Syt1 WT: 0.2142 ± 0.0885 nA, n=19; Syt1 9Pro: 0.3219 ± 0.172 nA, n=26), 20Hz (Syt1 WT: 0.7219 ± 0.367 nA, n=19; Syt1 9Pro: 0.6947 ± 0.335 nA, n=26), and 40Hz (Syt1 WT: 0.8852 ± 0.474 nA, n=19; Syt1 9Pro: 0.9623 ± 0.505 nA, n=26), and in Syt1 WT and 9Pro expressing synapses. All recordings at room temperature, unmonitored. (* $p < 0.05$, Linear regression model with interaction effects).

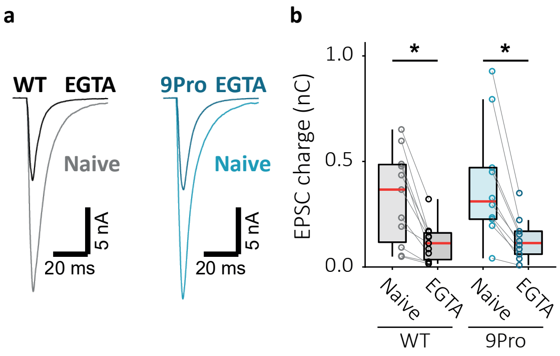

Supplementary Figure S7. EGTA-AM affects single EPSCs similarly in Syt1 WT and 9Pro expressing synapses

(a) Single EPSC representative traces and **(b)** boxplot of charge transferred per EPSC in Syt1 WT and 9Pro expressing synapses in the absence (Naive) and presence of EGTA-AM (WT Naive: 0.3666 ± 0.175 nC; WT EGTA-AM: 0.1125 ± 0.0545 nC, n=11; 9Pro Naive: 0.3107 ± 0.127 nC; 9Pro EGTA-AM: 0.1133 ± 0.0543 nC, n=10). All recordings at room temperature, unmonitored. (* $p < 0.05$, Wilcoxon signed-rank test).

Supplementary Figure S8

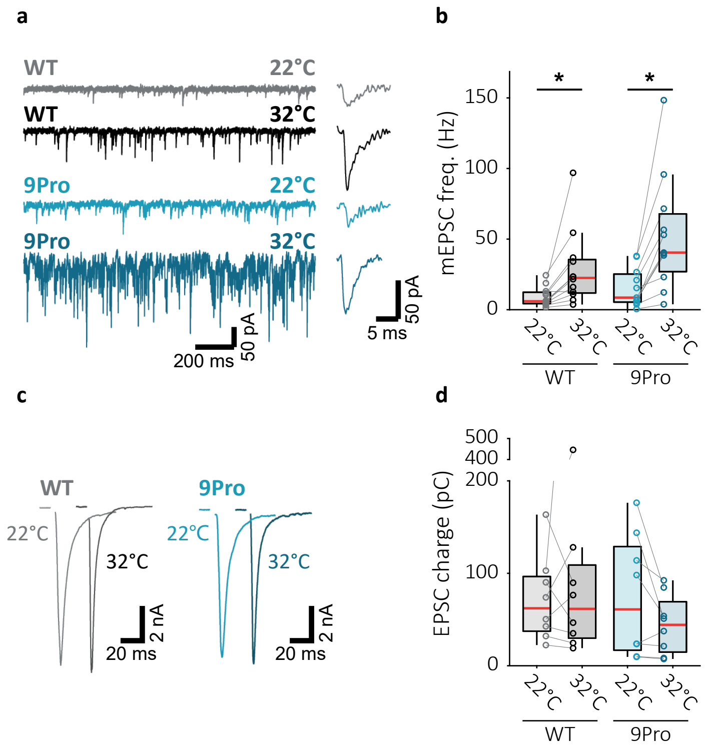

Supplementary Figure S8. Increased temperature has similar effects on spontaneous release and first evoked responses in Syt1 WT and 9Pro expressing synapses

(a) Representative traces of mEPSC recordings from Syt1 KO neurons rescued with WT (top) or 9Pro mutant (bottom) constructs at 22°C (light) and 32°C (dark) and **(b)** boxplot showing mEPSC frequency (WT 22°C: 5.993 ± 3.56 Hz; WT 32°C: 22.50 ± 11.56 Hz, n=12; 9Pro 22°C: 8.563 ± 6.82 Hz; 9Pro 32°C: 40.46 ± 17.4 Hz, n=11). (c) Single EPSC representative traces and **(d)** boxplot of charge transferred per EPSC at 22°C and 32°C in Syt1 WT and 9Pro expressing synapses (WT 22°C: 62.18 ± 29.1 pC; WT 32°C: 61.54 ± 32.6 pC, n=8; 9Pro 22°C: 60.97 ± 51.16 pC; 9Pro 32°C: 44.27 ± 29.5 pC, n=8). (* $p < 0.05$, Wilcoxon signed-rank test).
